# Genetic expression and mutational profile analysis in different pathologic stages of hepatocellular carcinoma patients

**DOI:** 10.1186/s12885-021-08442-y

**Published:** 2021-07-08

**Authors:** Xingjie Gao, Chunyan Zhao, Nan Zhang, Xiaoteng Cui, Yuanyuan Ren, Chao Su, Shaoyuan Wu, Zhi Yao, Jie Yang

**Affiliations:** 1 Department of Biochemistry and Molecular Biology, Department of Immunology, School of Basic Medical Sciences, Tianjin Medical University, Tianjin, China; 2 Key Laboratory of Immune Microenvironment and Disease, Ministry of Education, Key Laboratory of Cellular and Molecular Immunology in Tianjin, Excellent Talent Project, Tianjin Medical University, Tianjin, China; 3 Department of Neurosurgery Ministry of Education and Tianjin Municipal Government Laboratory of Neuro-Oncology Key Laboratory of Neurotrauma, Variation, and Regeneration , Tianjin Neurological Institute Tianjin Medical University General Hospital , Tianjin, China

**Keywords:** Expression, Mutation, HCC, Pathologic stage, Prognosis

## Abstract

**Background:**

The clinical pathologic stages (stage I, II, III-IV) of hepatocellular carcinoma (HCC) are closely linked to the clinical prognosis of patients. This study aims at investigating the gene expression and mutational profile in different clinical pathologic stages of HCC.

**Methods:**

Based on the TCGA-LIHC cohort, we utilized a series of analytical approaches, such as statistical analysis, random forest, decision tree, principal component analysis (PCA), to identify the differential gene expression and mutational profiles. The expression patterns of several targeting genes were also verified by analyzing the Chinese HLivH060PG02 HCC cohort, several GEO datasets, HPA database, and diethylnitrosamine-induced HCC mouse model.

**Results:**

We identified a series of targeting genes with copy number variation, which is statistically associated with gene expression. Non-synonymous mutations mainly existed in some genes (e.g.,*TTN*, *TP53*, *CTNNB1*). Nevertheless, no association between gene mutation frequency and pathologic stage distribution was detected. The random forest and decision tree modeling analysis data showed a group of genes related to different HCC pathologic stages, including *GAS2L3* and *SEMA3F*. Additionally, our PCA data indicated several genes associated with different pathologic stages, including *SNRPA* and *SNRPD2*. Compared with adjacent normal tissues, we observed a highly expressed level of *GAS2L3*, *SNRPA*, and *SNRPD2* (*P* = 0.002) genes in HCC tissues of our HLivH060PG02 cohort. We also detected the high expression pattern of *GAS2L3*, *SEMA3F*, *SNRPA*, and *SNRPD2* in the datasets of GSE102079, GSE76427, GSE64041, GSE121248, GSE84005, and the qPCR assay using diethylnitrosamine-induced HCC mouse model. Moreover, SEMA3F and SNRPD2 protein were highly stained in the HCC tissues of the HPA database. The high expression level of these four genes was associated with the poor survival prognosis of HCC cases.

**Conclusions:**

Our study provides evidence regarding the gene expression and mutational profile in different clinical pathologic stages of TCGA HCC cases. Identifying four targeting genes, including *GAS2L3*, *SNRPA*, *SNRPD2,* and *SEMA3F*, offers insight into the molecular mechanisms associated with different prognoses of HCC.

**Supplementary Information:**

The online version contains supplementary material available at 10.1186/s12885-021-08442-y.

## Background

Several factors (e.g., genetic, epigenetic alteration, immune microenvironment, hepatitis B/C virus infection) contribute to the progression, diagnosis, and prognosis of hepatocellular carcinoma, the primary histological subtype of liver cancer [1–4]. The pathologic stages (I, II, III, IV) of HCC are closely related to the clinical prognosis of liver cancer [5, 6]. The radical therapies, such as resection, radiofrequency ablation, or transplantation, are often valid and feasible for the HCC patients with early pathologic stage [7, 8]. It is therefore meaningful to identify the potential pathologic stage-related genes of HCC.

The TCGA (The Cancer Genome Atlas) database stores the multiple-genomics data from more than 13 types of cancer, such as gene expression, CNV (copy number variation), SNV (simple nucleotide variation), SNP (single nucleotide polymorphism), and clinical information (http://tcga-data.nci.nih.gov/tcga/) [9, 10]. There are more than 360 HCC cases within the TCGA-LIHC (liver hepatocellular carcinoma) cohort, and the corresponding expression/mutation matrix and clinical features are available. As another public data repository, the GEO (Gene Expression Omnibus) database of NCBI (National Center for Biotechnology Information) also contains a series of available functional genomics datasets for different types of clinical diseases (https://www.ncbi.nlm.nih.gov/geo/) [11]. The HPA (Human Protein Atlas) database contains various types of human proteomic datasets, such as mass spectrometry-based proteomics or immunohistochemistry images (https://www.proteinatlas.org/about) [12].

In the present study, we first conducted the statistical analysis, random forest, decision tree, and principal component analysis to identify the differential gene expression, CNV, SNV, and SNP profiles linked to the HCC pathologic stages within the TCGA-LIHC cohort. Furthermore, we confirmed the expression feature and prognostic value of several novel targeting genes, using our Chinese HLivH060PG02 HCC cohort, the Diethylnitrosamine-induced HCC mouse model, the available datasets of TCGA, GEO, and HPA database, respectively.

## Methods

### HCC pathologic stage-associated gene analysis

We first downloaded the liver cancer-associated mRNA, lncRNA expression matrix with the workflow type of “HTSeq-Counts” and clinical data from the TCGA-LIHC cohort, using a “TCGAbiolinks” R package. Then, the clinical information (e.g., gender, age, race, ethnicity, height, weight, clinical pathologic T/N/M stage, neoplasm histologic grade, survival status, follow-up time, and various clinical, biochemical indicators) was extracted. Three groups of clinical pathologic stages (I, II, and III-IV) were investigated. We performed the Kruskal-Wallis test or chi-square test to analyze the correlation between the pathologic stages and the clinical indicators of HCC cases through GraphPad Prism software (San Diego, California, USA). Also, we performed a series of logrank test and KM (Kaplan-Meier) survival curve analyses using SPSS 20.0 statistical analysis software.

The expression matrix and clinical feature information were merged, and the non-HCC case data were excluded, using R language software (https://www.r-project.org/). We then used an “EdgeR” package for the followed TMM data standardization and differential gene screening work. Logarithm base 2 (log 2)-treated gene expression matrix was applied. The volcano maps were generated by a “ggplot” R package. Based on an online venn tool (http://bioinformatics.psb. ugent.be/webtools/Venn/), an intersection analysis was performed to obtain the common genes of different groups. Then, Morpheus online software (https://software.broadinstitute.org/Morpheus/) was applied to obtain a heat map of cluster analysis. Gene ID conversion was implemented by a conversion tool of DAVID (database for annotation, visualization and integrated discovery; https://david.ncifcrf.gov/conversion.jsp). We performed a protein-protein interaction network analysis of common genes through a STRING online analysis tool (https://string-db.org/). The expression pattern among the groups of total HCC, negative control, stage I, stage II and stage III-IV, and the prognostic survival value of target genes were analyzed by a web server GEPIA2 (gene expression profiling and interactive analyses, version two; http://gepia2.cancer-pku.cn/#index) [13, 14].

### Copy number variation analysis

The CNV datasets with the type of masked copy number segment within the TCGA-LIHC cohort were downloaded from the TCGA database. Based on the CNV chromosome location information, the corresponding gene annotations were added by the Perl script. Segment_mean value between − 0.2 and + 0.2 was considered as no variation and marked as “0”. There were the CNV types of the double deletion (dd, “-2”), single deletion (sd, “-1”), single gain (sg, “+ 1”), and amplication (A, “+2 or +>2”). We obtained the CNV differential targeting genes between HCC and the normal control group by a chi-square test and the Bonferroni-adjusted *P* value correction method. Circos 2D track plot was generated by a “RCircos” R package.

After combining gene expression matrix and CNV differential targeting gene data, a Kolmogorov-Smirnov test for correlation analysis was performed to identify the expression-correlated targeting genes with CNV. Then, the “enrichGO” function was applied for a GO (Gene Ontology) analysis, while “enrichKEGG” function was for a KEGG (Kyoto Encyclopedia of Genes and Genomes) analysis. Finally, we constructed a PPI (protein-protein interaction) network using a “STRINGdb” R package and identified key hub genes within the PPI network using a “Molecular Complex Detection” (MCODE) modular analysis of cytoscape software.

### Random forest and decision tree analysis

After merging the above clinical information, mutation, and expression matrix, we performed a random forest modeling analysis using a “randomForest” R package. The specific gene profiles of normal controls, overall HCC cases, and HCC cases with different pathologic stages were effectively classified by the principles of “mean decrease accuracy” and “mean decrease Gini”. The result was visualized by a “ggpubr” R package. Multi dimension scale plot was obtained by a “MDSplot” function. Using the “pROC” R package, ROC (receiver operating characteristic) curves were plotted, and the AUC (area under the ROC curve) value was calculated. Moreover, we performed a decision tree modeling analysis using “rpart” and “rpart.plot” R packages.

### Genetic mutational analysis

From the TCGA-LIHC cohort, we directly downloaded the SNV data with the type of masked somatic mutation and extracted the mutation matrix using the Perl script. Based on the mutation rate, the top 15 genes were selected, and the “GenvisR” R package was utilized to draw a waterfall map containing the clinical stage information. Also, we extracted the SNP data, and performed a wilcox test to analyze the correlation between gene mutation and expression in overall HCC and different pathologic stages. The data was visualized by a “boxplot” function. We further used a “survminer” R package to correlate the specific gene mutations and the clinical prognosis and performed logrank test and KM survival curve analyses to drew the corresponding survival curves.

### Principal component analysis

To identify the HCC pathologic stage-associated genes of TCGA-LIHC, we performed a principal component analysis (PCA) using the “prcomp” function. The principal component (PC) gravity and gene contribution maps were obtained by two R packages of “factoextra” and “ggplot2”. A three-dimensional map (PC1, PC2, and PC3) was drawn using a “scatterplot3d” R package; while a two-dimensional map (PC1, PC2) was generated through a “ggord” R package. Additionally, for specific genes selected by a decision tree, random forest, and principal component analysis, we applied the R language to obtain the expression matrix of overall HCC tissue and adjacent normal tissue and performed a wilcox.test using GraphPad Prism software. Also, we analyzed the expression pattern of these genes among stage I, II, III and IV using the “Stage Plot” modules of GEPIA2 (http://gepia2.cancer-pku.cn/#analysis) [13, 14].

### Chinese HLivH060PG02 HCC cohort analysis

Besides the above TCGA-LIHC cohort, we also utilized the datasets of a Chinese HLivH060PG02 HCC cohort (Shanghai Outdo Biotech Co., Ltd., Shanghai, China). The main clinical characteristics of HCC cases were shown in Additional file 1: Table S1, and the use of human biological materials (Number: YB M-05-02) was approved by the Use Ethics Committee of Shanghai Outdo Biotech Company. We detected the expression difference of five targeting genes (*GAS2L3*, *CUZD1*, *SNRPA*, *SNRPD2*, *SEMA3F*) between 30 HCC tissues and corresponding adjacent normal tissues. The correlation of gene expression with pathologic stages was also analyzed. Based on an ABI 7500 Real-Time PCR System (Thermo Fisher Scientific), a quantitative real-time PCR (qPCR) assay was performed with a TB Green™ Premix Ex Taq™ II (Takara, RR820A). Primer sequences: *GAS2L3* [5′-CTGAGGACCCTCCTTGTAGTTG-3′ (Forward, F), 5′-CCTTGAAGAGTATCCCAGCCTC-3′ (Reverse, R)]; *CUZD1 *[5′-CCAGCCTTTCAACAGTGTGC-3′ (F), 5′-GCCACGAGGTAGCATTTCCT-3′ (R)]; *SNRPA* [5′-ACCCGCCCTAACCACACTAT-3′ (F), 5′-GGAGAAGATGGCGTACAGGG-3′ (R)]; *SNRPD2* [5′-CAAGTGCTCATCAACTGCCGCA-3′ (F), 5′-GCGGTCTTTGTTGACTGGCTTG-3′ (R)]; *SEMA3F* [5′-CAAGGATGTCAACGGCGAGT-3′ (F), 5′-TGAGTCTGGGTCCATGGTGT-3′ (R)]; *beta-actin* [5′-GAAGAGCTACGAGCTGCCTGA-3′ (F), 5′-CAGACAGCACTGTGTTGGCG-3′ (R)]. Finally, we performed a wilcox.test using GraphPad Prism software. Differences with *P* less than 0.05 were considered significant.

### GEO dataset verification

First, we utilized the “GeoQuery” R package to download the expression matrix and clinical information of GSE102079, GSE76427, GSE64041, GSE121248 and GSE84005 from the GEO database, respectively. After data sorting, “match” function was used to combine the expression matrix and paired clinical information. Then, referring to the published report [15], we utilized the “compare_means (paired = T)” function within the “ggpubr” R package to perform a wilcox.test. The results were finally visualized by the “ggdotchart” function of “ggpubr” package.

### DEN-induced HCC mouse model

At 2 weeks after birth, C57BL/6 mice (purchased from Academy of Military Medical Sciences, China) were intraperitoneally injected with 20 mg/kg saline solution containing DEN (Diethylnitrosamine) (N0258-1G, Sigma). After 48 weeks, the mice were sacrificed for dissection, and the tissue samples of HCC and adjacent non-tumor controls were obtained successfully (n = 14). Total RNA was then extracted and reverse-transcribed into cDNA using the RevertAid First Strand cDNA Synthesis Kit (K1622, Thermo Fisher). Finally, a qPCR was performed using an ABI-StepOne Plus (Life Technologies). Primer sequences: *GAPDH* [5′-CATCACTGCCACCCAGAAGACTG-3′ (F), 5′-ATGCCAGTGAGCTTCCCGTTCAG-3′ (R)]; *GAS2L3* [5′-GAGACCTTGCTTAATGCCTCGG -3′ (F), 5′-CGATGAGAGCAGCTACAAGGAG-3′(R)]. Then, we performed a wilcox.test by the “compare_means (paired = T)” function and visualized the data through a “‘ggplot2’” R package.

### Immunohistochemistry analysis

As reported previously [16], we logged into the HPA database (https://www.proteinatlas.org/pathology) to obtain the available immunohistochemistry analysis data of SNRPD2 and SEMA3F proteins in the normal liver and HCC tissues.

### Survival curve analysis

Targeting the four genes (*GAS2L3*, *SNRPA*, *SNRPD2*, *SEMA3F*), we utilized the “Survival Analysis” module of GEPIA2 to perform the survival curve analysis of OS (overall survival) and DFS (disease-free survival), respectively. The group cutoff of “Median” and axis units of “Months” were used. The plots with 95% confidence interval, *P* value of logrank test, HR (hazards ratio), and *P* value of Mantel-Cox test were generated. The survival curve analyses of two signatures, including “*SNRPA*/*SNRPD2*” and “*SNRPA*/*SNRPD2*/*GAS2L3*/*SEMA3F*”, were performed as well.

## Results

### HCC pathologic stages of TCGA-LIHC cohort

**Fig. 1:**
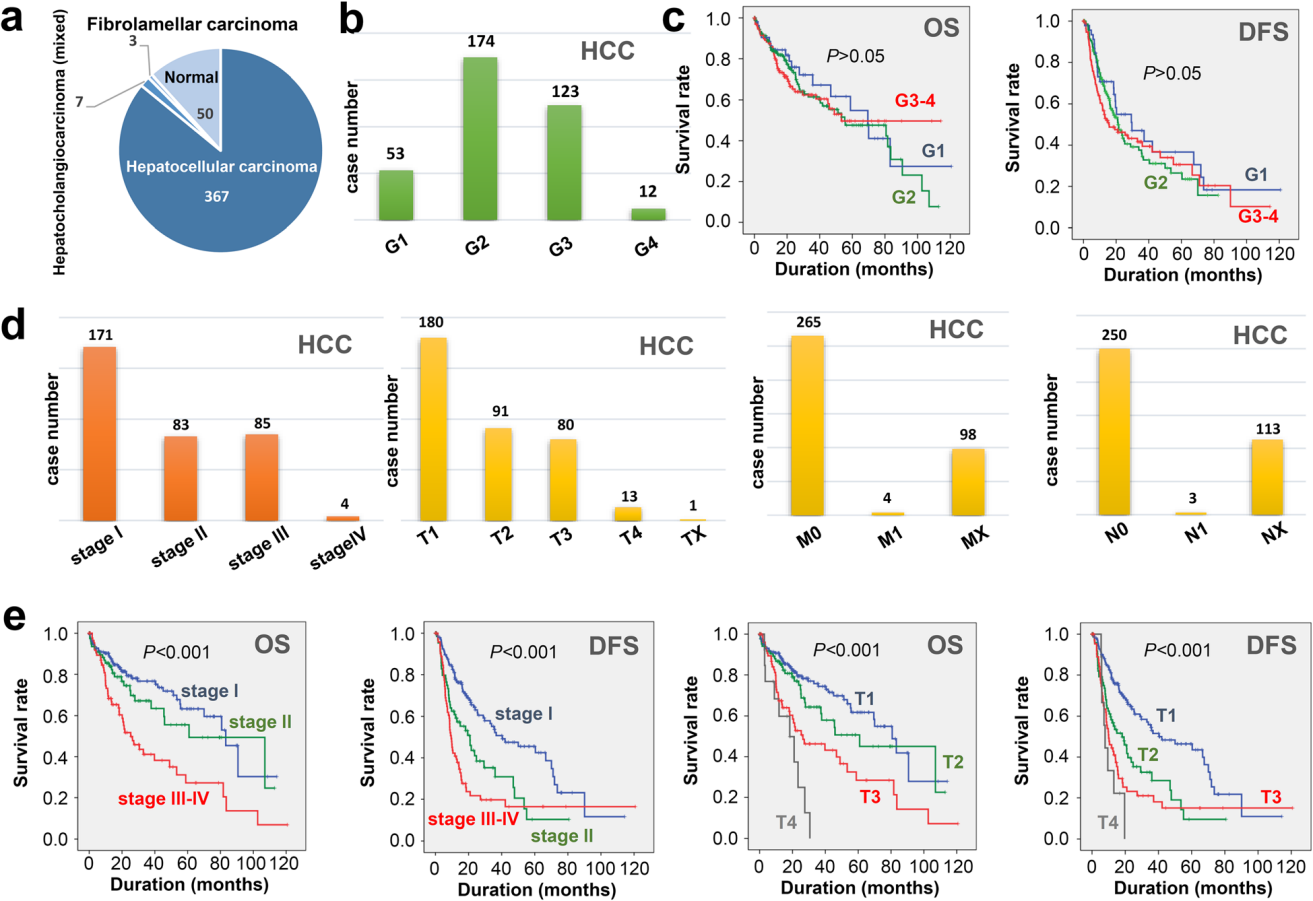
Survival curve analysis for different pathologic stages of HCC cases within TCGA-LIHC cohort. **a** Liver cancer cases and adjacent normal controls. **b** Neoplasm histologic grades (G1, G2, G3 and G4) of HCC cases. **c **Logrank test and KM survival curve analysis according to the histologic grades of HCC. **d** Clinical pathologic stages (stage I, stage II and stage III-IV) and T/N/M stage of HCC cases. **e** Survival curve analysis according to the stage I, II, III-IV and T1-T4.

From the TCGA-LIHC cohort, we extracted the expression matrix and clinical information of 367 hepatocellular carcinomas, three fibrolamellar carcinomas, seven hepatobiliary mixed carcinomas, and 50 adjacent normal controls (Fig. 1a). The correlation between the histologic grades of HCC (Fig. 1b, G1/G2/G3/G4) and clinical outcomes of HCC cases were analyzed. As shown in Fig. 1c, we did not detect a statistically significant difference in the survival assessment of OS/DFS among different histologic grades (*P* > 0.05). Figure 1d showed the detailed case number information regarding the clinical pathologic stages (stage I, II, III-IV) and TNM staging of HCC cases within the TCGA-LIHC cohort. As expected, stage III-IV or T4 patients showed the worst prognosis, whereas stage I or T1 patients had a better prognosis (Fig. 1e, *P* < 0.001).

Besides, we analyzed the association between different pathologic stages (stage I, II, III-IV) and clinical parameters. The total bilirubin, albumin, fetoprotein, and platelet count indicators, but not creatine and protherombin time, showed a statistical correlation with the different HCC pathologic stages (Additional file 2: Fig. S1 a-f, *P* < 0.05). Furthermore, we failed to observe a correlation between HCC pathologic stages and other factors, including age, height, weight, race, ethnicity, and gender (Additional file 2: Fig.S1g-l). In this study, we explored the gene expression and mutational profiles associated with different clinical pathologic stages of HCC cases within the TCGA-LIHC cohort.

### Differential gene screening

First, we tried to identify the genes that showed an increment or decrement trend in the normal, stage I, stage II, and stage III-IV groups. A range of differential genes for the three comparison groups, including Tumor vs. Normal, stage II vs. stage I, stage III + IV vs. stage II, were screened out. We showed the volcano plots of the above three sets in Additional file 3: Fig. S2a. Then, we conducted an intersection analysis of the up- and down-regulated genes. As shown in Additional file 3: Fig. S2b, we obtained a total of 12 up-regulated genes but no down-regulated genes. These genes did not establish the protein-protein interaction relationship and mainly existed in the stage III + IV, but not with a high proportion (Additional file 3: Fig. S2c-d). The full name information of these genes were listed in Additional file 3: Fig. S2e.

Next, we analyzed the expression patterns of these genes in normal, tumor, and different pathologic stages of HCC cases, respectively. As shown in Additional file 4: Fig. S3a-b, except *CRTAC1* gene, other genes showed a higher expression level in the tumor group compared with the normal controls. However, only the gene expressions of *DUOX2*, *IQCA1*, *PCSK1*, *HOXB9*, *KCNH2*, and *NPTX1* were statistically associated with the distribution of HCC stage I-IV. Further, the survival analysis results of the OS and DFS suggested that the highly expressed *CUZD1* and *IQCA1* were related to a poor prognosis of HCC cases (Additional file 4: Fig. S3c).

### Copy number variation analysis

**Fig. 2:**
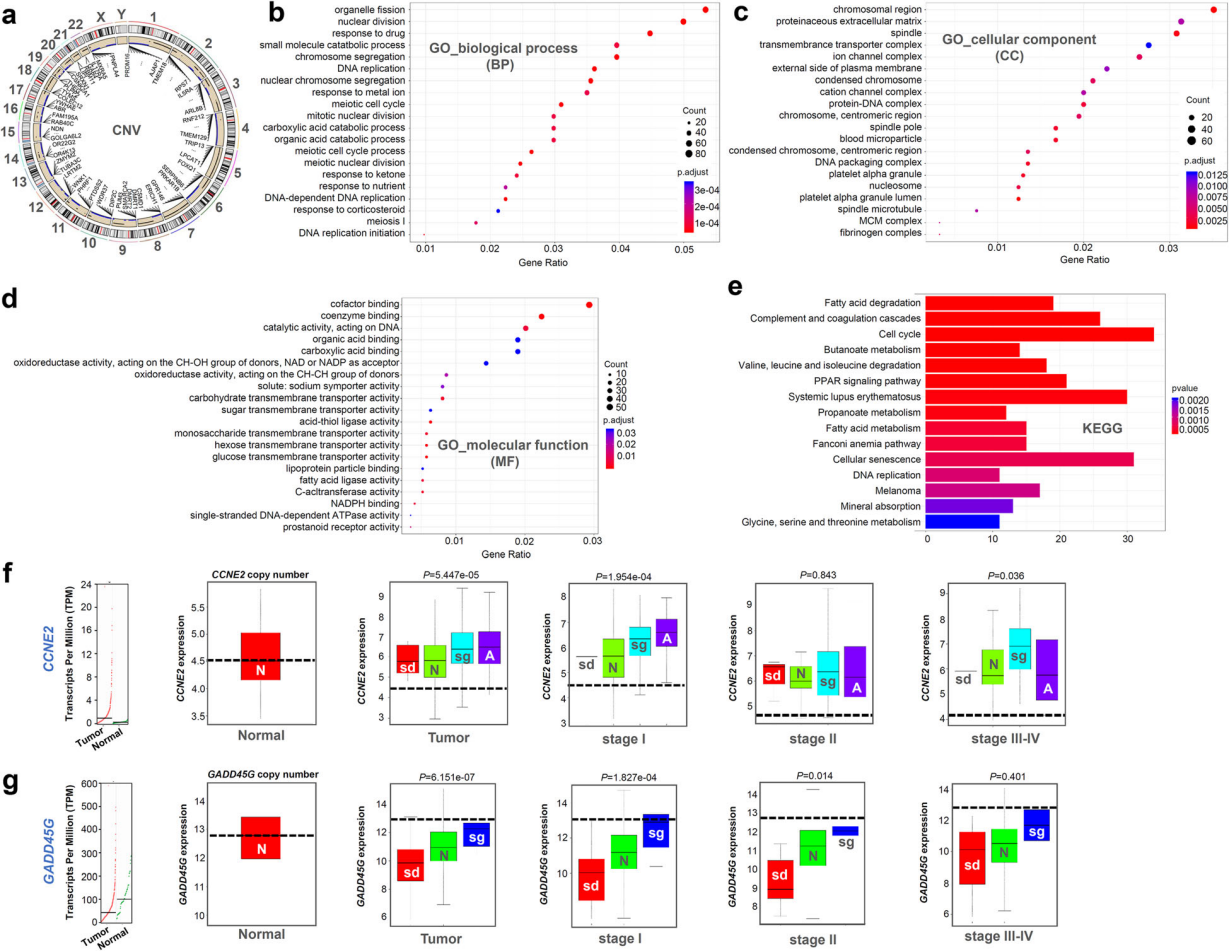
Genetic CNV analysis for different pathologic stages of HCC. **a** Circos 2D track plot of CNV profile. **b-e** GO and KEGG analysis data of the genes with CNV, which was correlated with gene expression. **f-g** Expression levels of *CCNE2*, *GADD45G* in normal and tumor by GEPIA2, and the correlation between gene expression and CNV in normal and different pathologic stages of HCC

We performed the somatic CNV analysis and identified a total of 16,644 genes with CNV from the TCGA-LIHC cohort. Circos 2D track plot for the CNV distribution in the chromosomes was shown in Fig. 2a. We then utilized a Kolmogorov-Smirnov test to analyze the correlation between CNV and gene expression and screened a group of genes. Our GO and KEGG analysis data further showed that most of these genes were implicated in cell division or the cell cycle (e.g., organelle fission, nuclear division, and spindle location) (Fig. 2b-e). For instance, the CNV of cell cycle-related *CCNE2 *gene in the groups of Tumor, stage I, and stage III-IV was statistically correlated with the gene expression (Fig. 2f). However, the *GADD45G* expression level in HCC cases was lower than that in the negative controls, hinting at the presence of other potential gene expression inhibition mechanisms (Fig. 2g). We presented some CNV-driven genes involved in the cell cycle pathway in Additional file 5: Fig. S4.

### Protein-protein interaction network analysis

**Fig. 3:**
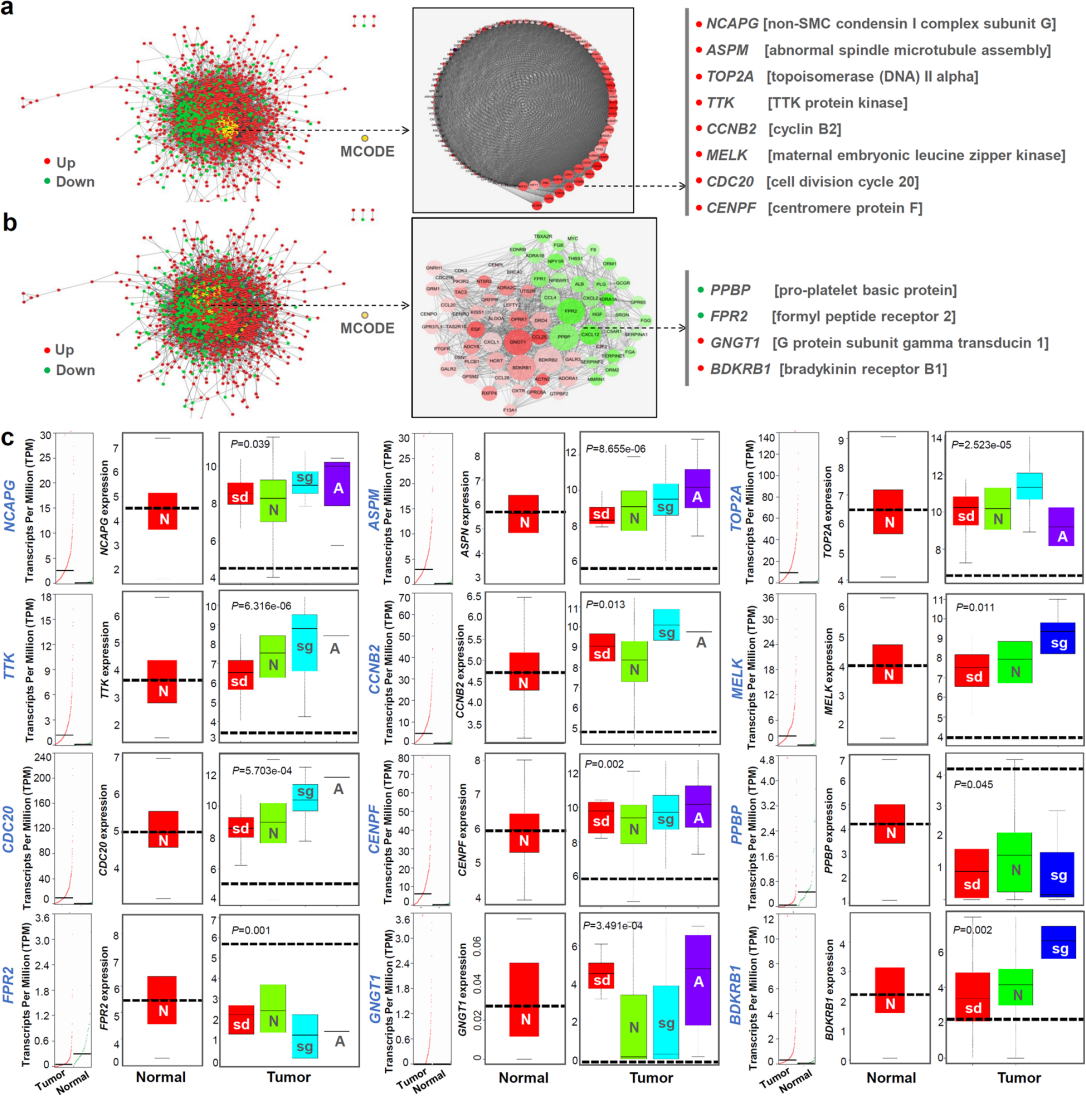
Protein-protein interaction network. **a-b** “STRINGdb” R package and cytoscape software, and MCODE were used for the construction of PPI network and the identification of hub genes. **c** Expression levels in normal and tumor, and the correlation between gene expression and CNV of some hub genes in normal and different pathologic stages of HCC were analyzed.

Targeting the above-identified genes, we built a protein-protein interaction (PPI) network and identified several hub genes. As shown in Fig. 3a-b, there were two modules with the highest ratings. The expression levels of identified hub genes were statistically related to the copy number variation. Of them, highly expressed cell cycle-related genes (e.g., *TTK*, *CDC20,* and *ASPM*) exhibited a significant positive correlation with copy number variation (Fig. 3c).

### Genetic mutation analysis

We downloaded the HCC-related SNV data and selected the top 15 genes with the most frequent mutation frequency (e.g.,*TTN*, *TP53*, *CTNNB1*, *MUC16*, and *ALB)* to map the waterfall with clinical stage information. As shown in Additional file 6:Fig. S5a, the gene mutation types were mainly non-synonymous mutations. The mutation frequency of 286 HCC cases with mutations was not related to the clinical pathologic stages of HCC (stage I, II, III-IV). Additionally, for *CTNNB1*, we observed the correlation between high expression and mutation status in overall HCC, stage I, II, III-IV groups (Additional file 6: Fig. S5b). *TP53* gene mutation was statistically linked to a reduced expression of *TP53* in the overall HCC, stage I, II groups (Additional file 6: Fig. S5b). However, *OBSCN* mutation was correlated with the low expression of *OBSCN* in overall HCC and specific stage I groups (Additional file 6: Fig. S5b).

We further performed a waterfall map analysis of the above-mentioned *CENPF*, *ASPM*, *MELK*, *TTK*, *GADD45G*, *CDC20*, *CCNE2,* and other interesting genes. We did not detect the association between the low mutation frequency of these genes and pathologic stages or gene expression, although non-synonymous mutations mainly existed (Additional file 7: Fig. S6). Also, we found that variations in the *CTNNB1*, *TP53*, *TTN*, and *OBSCN* genes were not related to the clinical prognosis of HCC cases with different pathologic stages (Additional file 8: Fig. S7; Additional file 9: Fig. S8).

Subsequently, we extracted the SNP data of HCC cases from the TCGA-LIHC cohort and found that the rs121913396, rs121913400, rs121913407 SNP of *CTNNB1,* and rs28934571 SNP of *TP53* gene were relatively high frequency (Additional file 10: Fig. S9a). There were more than 10 types of SNP for the *CTNNB1* gene (Additional file 10: Fig. S9b). Compared with the wild-type group, we observed a higher expression level for *CTNNB1* gene with rs121913396 and rs121913400 (Additional file 10: Fig. S9c). But there was still a lack of positive correlation between the rs121913396, rs121913400, rs121913407 of *CTNNB1* gene and the clinical prognosis of HCC (Additional file 11: Fig. S10a-c). Although there was no statistical correlation between *TP53* rs28934571 and gene expression (Additional file 10: Fig. S9c), we observed a worse prognosis of HCC cases with AA and CA genotypes of *TP53* rs28934571, compared with wild-type CC controls (Additional file 11: Fig. S10d).

### Random forest and decision tree analysis

We integrated the above clinical, mutation, and expression information to conduct a random forest modeling analysis. Multiple dimension scale plot in Additional file 12: Fig. S11a indicated an effective classification for the overall HCC cases and normal controls. AUC value of ROC equals 0.956, indicating a high classification accuracy (Additional file 12: Fig. S11b). We also provided the feature vectors extracted from the classification model in Additional file 12: Fig. S11c-d and identified the largely contributed genes (e.g.  *ECM1*, *FCN2*, *ANGPTL6*, *OIT3*, *ADAMTS13 *and *LRRC14*). Next, we performed a decision tree modeling analysis according to these genes. We first randomly selected 260 HCC cases for modeling, and then tested other 107 cases, and found that the predicted rates of the genes were larger than 90% (Additional file 12: Fig. S11e). Meanwhile, we analyzed the expression difference of these genes between 50 HCC tissues and adjacent non-tumor controls. There showed the higher expression levels of *ECM1*, *FCN2*, *ANGPTL6*, *OIT3 *and *ADAMST13* genes in overall HCC tissue, compared with that in control tissues (Additional file 12: Fig. S11f, *P* < 0.0001).

**Fig. 4:**
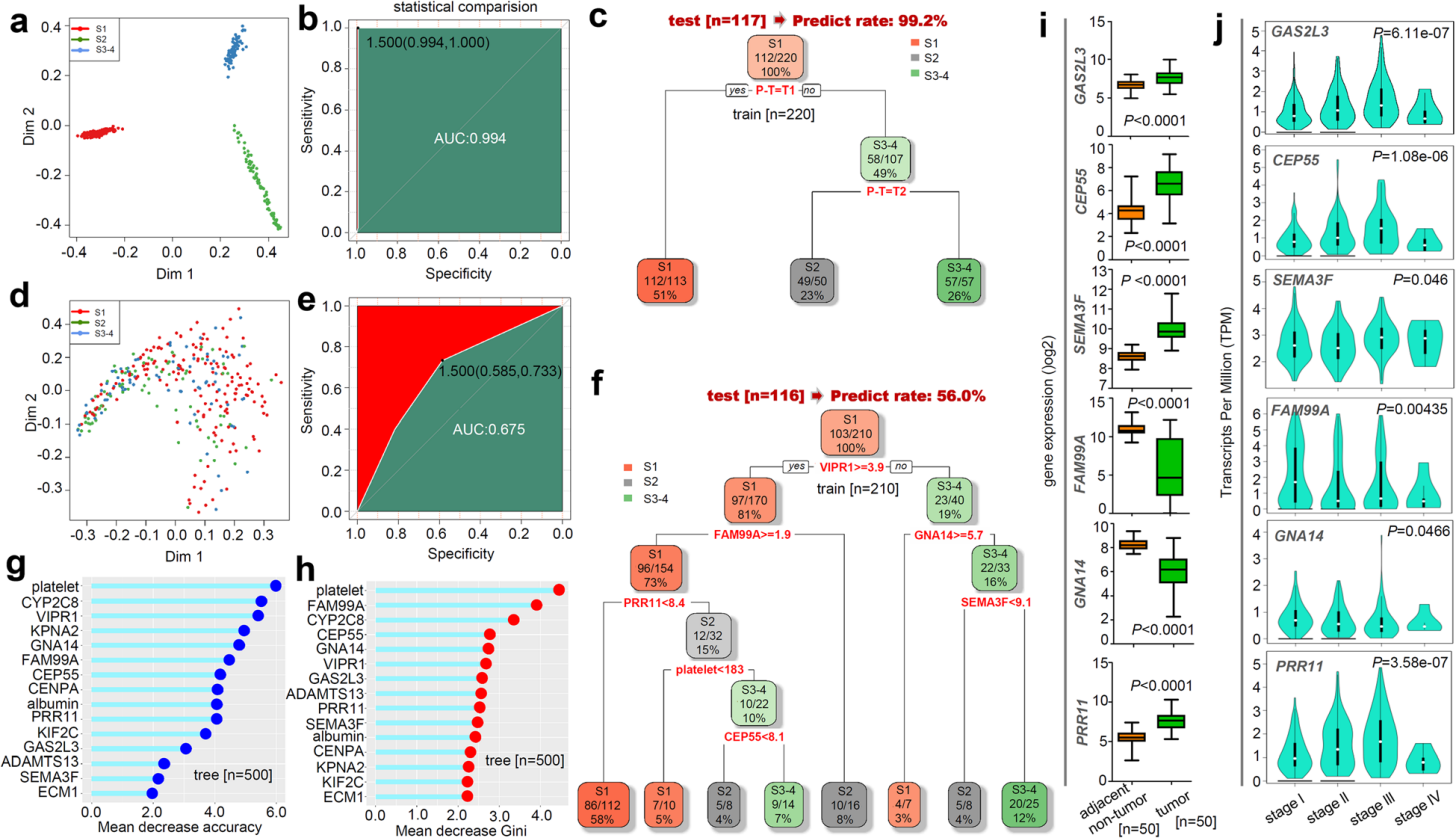
Decision tree and random forest analysis for different pathologic stages of HCC.** a** We combined the clinical, mutation and expression information to perform the random forest modeling analysis. Multiple dimension scale plot was provided. **b** ROC curve was plotted, and AUC value was calculated. **c** Decision tree modeling analysis was performed. **d-f** We excluded the TNM information to complete the random forest and decision tree modeling, again. **g-h** Based on the principles of mean decrease accuracy and mean decrease Gini, we identified the largely contributed genes. **i** Expression status of these genes in 50 HCC tissues with adjacent non-tumor tissues.** j** Expression data of stage I, II, III, and IV through the GEPIA2

We tried to build a random forest modeling with different HCC pathologic stages, which was closely related to the clinical TNM information. To prove the validity of this classification method, we conducted random forest and decision tree modeling analysis without removing TNM information. We found that T1 and T2 information could effectively distinguish stage I, II, III-IV with the AUC value of 0.994 in ROC and the prediction rate of 99.2% (Fig. 4a-c). Then, we excluded the TNM information for a new round of random forest modeling and observed a reduced classification effect (Fig. 4d-f, AUC = 0.675, predicting rate = 56.0%). Fig. 4g-h showed the genes that contributed significantly to the classification model. Compared with adjacent normal controls, *FAM99A *and *GNA14* genes were lowly expressed in the HCC tissues (Fig. 4i, *P* < 0.0001), whereas *GAS2L3*, *CEP55*, *SEMA3F*, and *PRR11* genes were highly expressed (*P* < 0.0001). Moreover, the expression levels of these genes were statistically associated with the different HCC pathologic stages (Fig. 4j).

### Principal component analysis

**Fig. 5:**
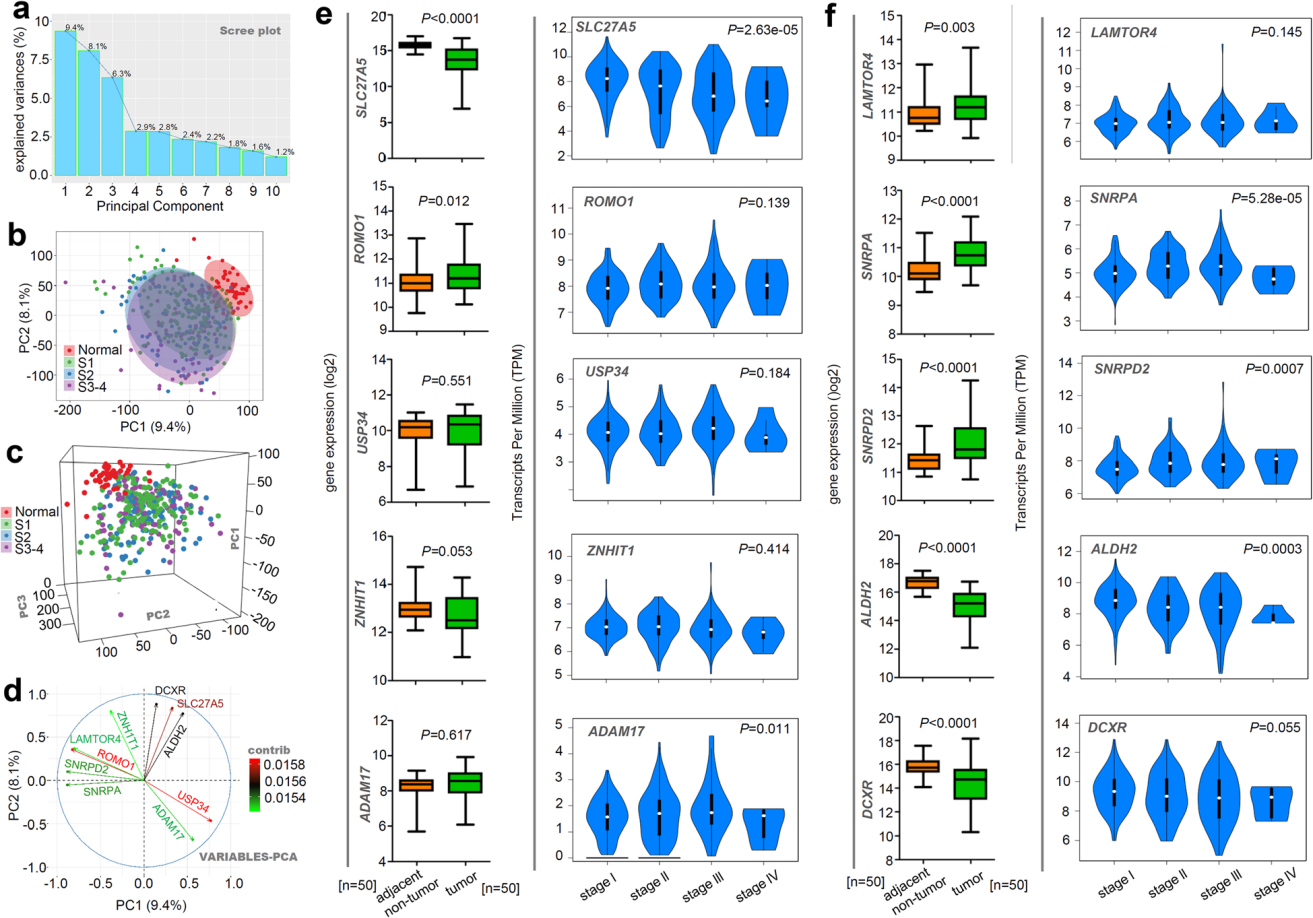
PCA analysis for different pathologic stages of HCC. **a** PC gravity. **b** Two-dimensional map (PC1/PC2). **c** Three-dimensional map (PC1/PC2/PC3). **d** Gene contribution map. **e-f** The expression levels of overall HCC, adjacent normal tissue, and stage I, II, III, and IV groups

Besides, we performed a principal component analysis to identify the target genes associated with different pathologic stages of HCC. As shown in Fig. 5a, the calculated variances of the PC1, PC2, and PC3 equaled 9.4, 8.1, and 6.3%, respectively. Based on the PC1/2 (Fig. 5b) and PC1/2/3 (Fig. 5c), we could effectively distinguish the normal controls and overall HCC cases, rather than the stage I, II, III-IV groups. Fig. 5d showed the top 10 genes that contributing mainly to PC1 and PC2. We analyzed the expression level of these genes between HCC tissue and adjacent normal tissue, or among different pathologic stages. As shown in Fig. 5e-f, compared with normal controls, the *SLC27A5*, *ALDH2*, and *DCXR* genes were lowly expressed (*P* < 0.0001), while *SNRPA* (*P* < 0.0001), *SNRPD2* (*P* < 0.0001), *LAMTOR4* (*P* = 0.003), *ROMO1* (*P* = 0.012) genes were highly expressed, in overall HCC tissues. Additionally, the expression of the *SNRPA*, *SNRPD2*, *SLC27A5*, *ADAM17*, and *ALDH2* genes was statistically related to the different HCC pathologic stages (Fig. 5e-f).

### Expression verification of targeting genes

**Fig. 6:**
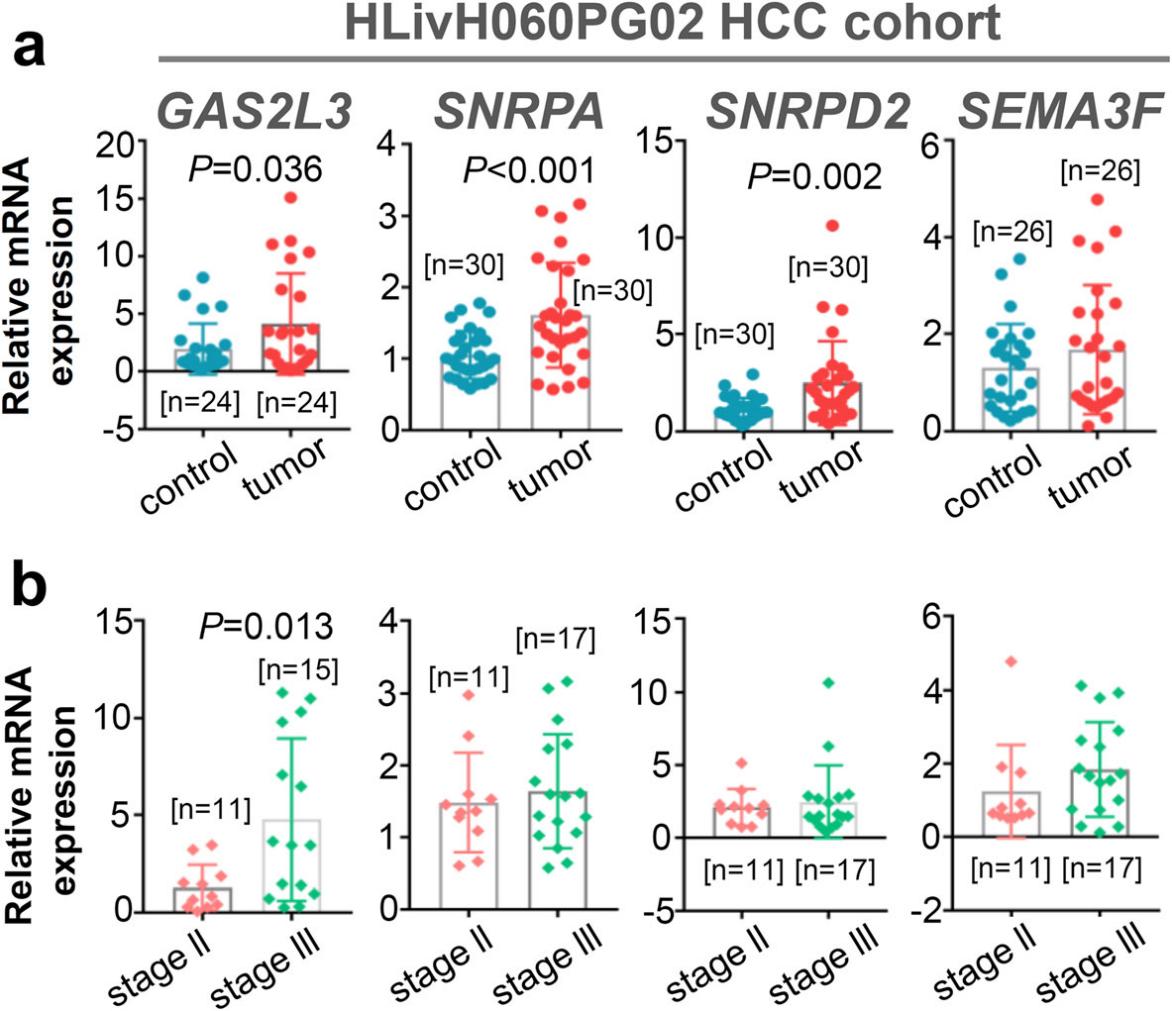
Expression levels of four targeting genes in HLivH060PG02 cohort. **a** We performed qPCR assay to detect the expression levels of *GAS2L3*, *SNRPA*, *SNRPD2*, and *SEMA3F* in Chinese HLivH060PG02 HCC cohort. **b** Correlation of gene expression and pathologic stages of HCC was analyzed as well. A wilcox.test was conducted

A series of HCC pathologic stage-associated genes were obtained through the above analyses of the TCGA-LIHC cohort. After the assessment of publication novelty through an online PubMed database retrieval, we further selected a total of six interesting genes, including *GAS2L3*, *SNRPA*, *SNRPD2*, *SEMA3F*, *IQCA1* and *OIT3*. We tried to verify the expression difference of these genes between the HCC tissues and adjacent normal tissues within our Chinese HLivH060PG02 HCC cohort. Unfortunately, due to the lower amplification efficiency of the *IQCA1* and *OIT3*, we finally selected the remaining four genes, namely *GAS2L3*, *SNRPA*, *SNRPD2*, and *SEMA3F*. Compared with adjacent normal tissues, we observed a highly expressed level of *GAS2L3* (Fig. 6a, *P* =0.036), *SNRPA* (*P* < 0.001), and *SNRPD2* (*P* = 0.002) genes in HCC tissues. Moreover, as shown in Fig. 6b, these genes in pathologic stage III showed a higher expression trend than in stage III, but statistical significance was only observed for the *GAS2L3* gene (*P* = 0.013).

**Fig. 7:**
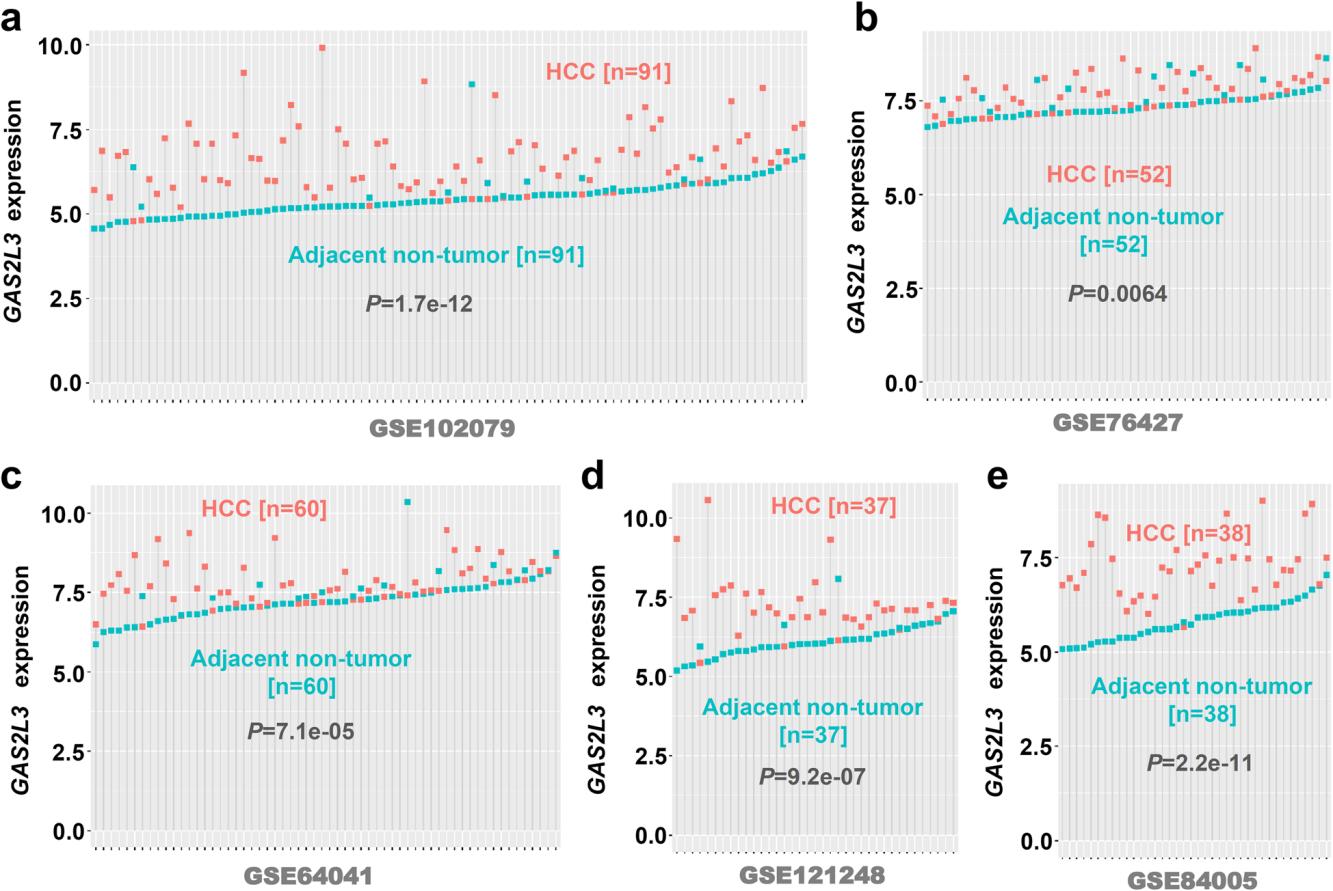
Expression level of *GAS2L3* in five GEO datasets. From the GEO database, we obtained five independent datasets, including **a** GSE102079 (n = 91), **b** GSE76427 (n = 52), **c** GSE64041 (n = 60), **d** GSE121248 (n = 37), **e** GSE84005 (n = 38), to analyze the expression difference of *GAS2L3* between HCC and adjacent non-tumor tissues. A wilcox.test was performed, and the results were visualized by “ggdotchart” function. Each vertical line represents one patient with HCC

**Fig. 8:**
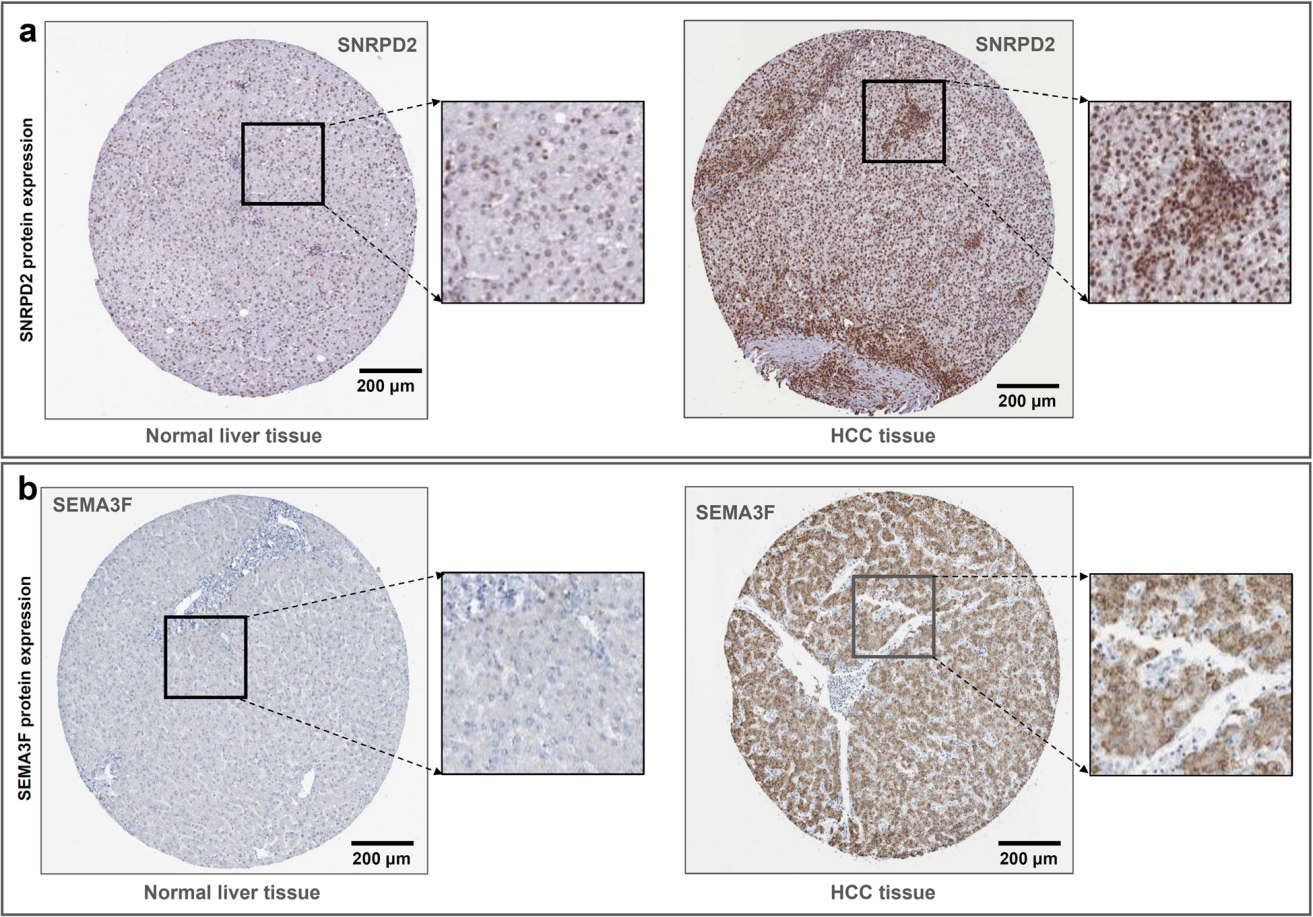
Immunohistochemistry staining of SNRPD2 and SEMA3F. Based on the available data of HPA database, the immunohistochemistry staining images of **a** SNRPD2 and **b** SEMA3F proteins in the normal liver and HCC tissues were provided. Bar, 200 μm.

To further confirm the high expression feature of these genes, we downloaded five available datasets (GSE102079, GSE76427, GSE64041, GSE121248, GSE84005), which containing the expression matrix between clinical tumor and adjacent non-tumor tissues. As shown in Fig. 7, Additional file 13: Fig. S12, and Additional file 14: Fig. S13, we observed the obviously high expression level of *GAS2L3*, *SNRPA, SNRPD2* in the HCC tissues, compared with adjacent non-tumor controls (all *P* < 0.05). The qPCR analysis using the DEN-induced HCC mice model also confirmed the high expression status of *GAS2L3* in HCC tissue (Additional file 15: Fig. S14, *P* = 0.0006 n = 14). Based on the available data from the HPA database, we observed the high expression of SNRPD2 in tumor tissues compared with normal liver tissue (Fig. 8a).

Regarding *SEMA3F*, we observed a higher expression trend of the *SEMA3F* gene in HCC and stage III groups, compared with the control group, although non statistical difference (Fig. 6a-b). Also, we observed the highly expressed *SEMA3F* in HCC tissues in the datasets of GSE102079, GSE64041, GSE121248, GSE84005 (Additional file 16: Fig. S15, all *P* < 0.05), only apart from the GSE76427 (*P* = 0.12). Besides, there existed a stronger staining signal of SEMA3F protein in the HCC tissues than the normal liver tissues (Fig. 8b).

### Survival curve analysis of target genes

**Fig. 9:**
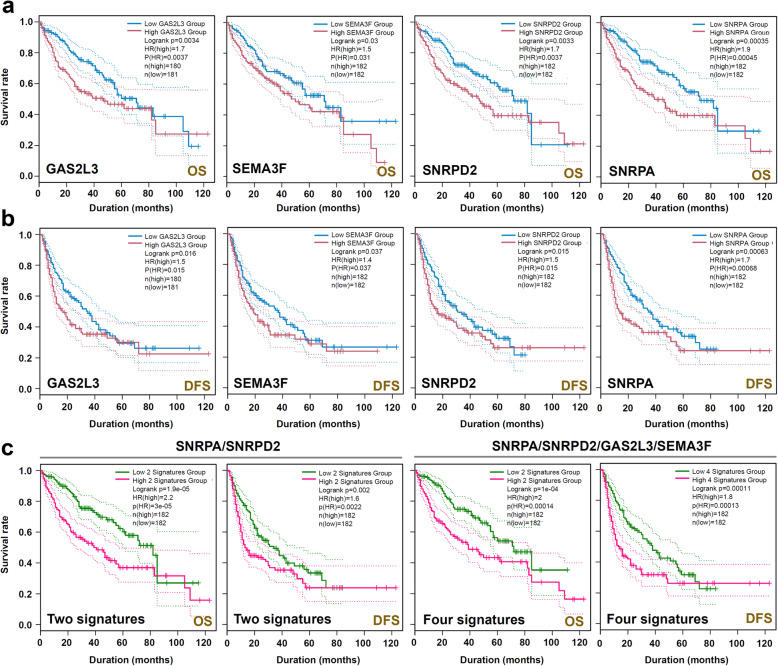
Survival curve analysis of *GAS2L3*, *SNRPA*, *SNRPD2*, *SEMA3F*. Targeting the four genes (*GAS2L3*, *SNRPA*, *SNRPD2*, *SEMA3F*), we utilized the GEPIA2 tool to perform the survival curve analysis of **a** OS and **b** DFS. **c** We also analyze the potential prognostic value of two signatures, including “*SNRPA*/*SNRPD2*” and “*SNRPA*/*SNRPD2*/*GAS2L3*/*SEMA3F*”

The survival analysis of OS and DFS further indicated the correlation between high expression levels of *GAS2L3*, *SNRPA*, *SNRPD2*, *SEMA3F *with the poor clinical prognosis (Fig. 9a-b, all *P* < 0.05, HR > 1). Furthermore, we observed the potential prognosis values of two signatures, including “*SNRPA*/*SNRPD2*” and “*SNRPA*/*SNRPD2*/*GAS2L3*/*SEMA3F*”, for the HCC cases (Fig. 9c, all *P* < 0.05, HR > 1).

## Discussion

Considering the complexity of etiology and pathogenesis of liver cancer, it is essential to continuously identify the potential oncogenes closely related to the pathogenesis of liver cancer. Based on the expression, mutation, and clinical evidence of liver cancer cases within the TCGA-LIHC cohort, we attempted to identify the potential liver cancer-related oncogenes. It should be noted that the TCGA-LIHC cohort includes not only HCC cases but also a tiny amount of fibrolamellar carcinomas and hepatobiliary mixed carcinomas cases. Considering the differences of distinct liver cancer types and limitation of sample sizes, we finally selected the cases of HCC for investigation. There are still very limited reports regarding differential gene expression, CNV, SNV, and SNP profiles from the points of different clinical pathologic stages (stage I, II, III, IV) and histologic grades (G1, G2, G3 and G4) of HCC cases within the TCGA-LIHC cohort, although several publications from other aspects or with different analysis strategies were retrieved [17–20]. We observe a statistical correlation between clinical outcomes of HCC cases and the pathologic stages, but not the histologic grades. Thus, we were interested in performing the genetic expression and mutational profile analysis in different pathologic stages of HCC cases.

Considering the small sample sizes, we combined the data of stage III and IV and focused on identifying differentially expressed genes associated with normal, stage I, stage II, and stage III-IV classifications. We first utilized the “EdgeR” package for the statistically significant differential genes in three comparisons (Tumor vs. Normal, stage II vs. stage I, stage III + IV vs. stage II), and further screened out the common genes. We did not identify the target genes with a decreasing trend in the groups of normal, stage I, stage II and stage III + IV), but several genes with an increasing trend and low expression frequency in stage III-IV. Thus, this strategy did not work well. Then, we utilized a PCA approach [15, 21, 22] to reduce the dimensionality of the datasets for the groups of normal, stage I, stage II, and stage III-IV, and to identify the genes that contributed largely to the main component. It was found that the PC1/2/3 could better distinguish the normal and tumor groups, but not the groups of stage I, stage II, and stage III-IV, may due to the low sample sizes and the complexity of different pathologic staging mechanisms of HCC. Despite this, we obtained the top 10 genes that mainly contributed to the PC1 and PC2. Of them, the expression levels of *SNRPA* and *SNRPD2,*two U1 snRNP component genes (*SNRPA* and *SNRPD2*) [23], were significantly associated with different HCC pathologic stages. Apart from PCA, we applied the random forest, a robust classification and regression approach [15, 24, 25], for the classification analysis of normal, tumor and stage I, stage II, stage III-IV groups. Although the classification effect for stage I, II, III-IV was worse than that for normal/tumor, we identified some critical contributing genes as well. Of them, *GAS2L3 *and *SEMA3F *gene was targeted.

Our data of the Chinese HLivH060PG02 HCC cohort, several GEO datasets, HPA database further confirmed the high expression status and potential clinical predictive value of *SNRPA*, *SNRPD2*, *GAS2L3 *and *SEMA3F* gene in HCC tissues. However, there still lack the molecular mechanism explored in-depth regarding the potential role of these genes in the tumorigenesis of HCC. In particular, GAS2L3, a member of GAS2 (growth arrest-specific 2) protein family, is associated with cell division [26]. We have established a DEN-induced HCC model, and observed the high expression of *GAS2L3 *in HCC tissues of mice. For the correlation of *GAS2L3* and HCC, only one study reported that *GAS2L3* could work as a member of six gene prognosis signature for the OS prediction of HCC cases [27]. Very recently, we reported that the high expression of *GAS2L3* is closely related to an enhanced proliferation and migration of glioma cells [28]. The potential role of *GAS2L3* in the oncogenesis of HCC merits more experiments.

Genetic CNV refers to the genome rearrangement-induced the copy number amplification or deletion of a large genome fragment (> 1 kb) [29, 30]. CNV-induced the gene expression alteration works as anessential mechanism of tumorigenesis [31, 32]. Herein, we utilized an analysis strategy to identify the interesting genes with CNV that are related to gene expression and clinical HCC pathologic stages within TCGA-LIHC cohort. We found that a group of cell cycle or cell division-associated genes with CNV. Additionally, we utilized MCODE modular analysis to screen out some key genes from the perspective of protein binding, which were also linked to cell cycle and division behavior. It is worth noting that the expression levels of some down-regulated genes in HCC (e.g., *GADD45G*, *FPR2*, *PPBP*) were closely linked to the CNV in a dose-dependent manner. Apart from CNV, some other key inhibition mechanisms of gene expression, such as hypermethylation modification, may exist for these genes.

Genetic mutation is considered the critical mechanism of tumorigenesis [33], and single nucleotide polymorphism is closely linked to the susceptibility of HCC in the population [34]. We performed a series of gene mutation alanalyses as well. We found that non-synonymous mutation was the main mutation type of these genes, and the gene mutation frequency was not statistically associated with the HCC pathologic stages. Although a correlation between the overall variation and expression of *CTNNB1* and *TP53* genes in HCC and different pathologic stages was observed, we failed to obtain the positive results for the mutations of a specific site in HCC cases with limited sample sizes. Additionally, there were more than 10 SNPs with low frequency for the *CTNNB1* gene in HCC cases. We also did not observe the correlation between these SNPs and *CTNNB1* high expression or clinical prognosis of HCC cases. More HCC cases may be required to validate this point.

## Conclusion

Taken together, we first utilized different bioinformatic approaches to provide the differential gene expression, CNV, SNV, and SNP profiles, which are associated with the different pathologic stage I, II and III-IV of HCC cases within the TCGA-LIHC cohort. Importantly, we identify four targeting HCC pathologic stage-associated genes, including *GAS2L3*, *SNRPA*, *SNRPD2* and *SEMA3F*. Compared with adjacent non-tumor tissues, these four genes were highly expressed in HCC tissues, presenting prognostic value for the HCC patients. More clinical sample tests are needed to determine whether the identified genes serve as the prognostic biomarker or therapeutic targets of HCC. The underlying molecular mechanisms merit further biology experiment evidence.

## Supplementary Information

**Additional file 1: Table S1.** Clinical characteristics of HCC cases in HLivH060PG02 cohort.

**Additional file 2: Figure S1.** Correlation between clinical or distribution characteristics and different pathologic stages of HCC. We performed the Kruskal-Wallis tests to analyze the relationship between pathologic stage I, II, III-IV, and **a** total bilirubin, **b** albumin, **c** fetoprotein, **d** creatine, **e** protherombin time, **f** platelet count, **g** age, **h** height and **i** weight, respectively. We also performed the chi-square tests to analyze the association between the factors of the **j** race, **k** ethnicity, **l** gender, and pathologic stage I, II, III-IV. * *P* < 0.05.

**Additional file 3: Figure S2.** Genetic difference analysis for different pathologic stages of HCC. **a** Volcano plots of Tumor vs. Normal, stage II vs. stage I, stage III + IV vs. stage II. **b** Intersection analysis of the above comparisons. **c** Protein-protein interaction network analysis of intersected genes. **d** A heat map of cluster analysis. **e** Full name information of the intersected genes.

**Additional file 4: Figure S3.** Expression and relative survival curve analyses for some targeting genes. **a** We analyzed the expression levels of *PIANP, DUOX2, CUZD1, CRTAC1, IQCA1, IL11, NPHS1, PCSK1, CPA1, HOXB9, KCNH2* and *NPTX1* genes in a normal and overall HCC, and **b** different pathologic stages by GEPIA2. **c** We also performed the Kaplan-Meier estimates of OS or DFS, according to the expression level.

**Additional file 5: Figure S4.** Comparison between CNV and expression level of genes within cell cycle pathway. We performed a Kolmogorov-Smirnov test for correlation analysis to identify the expression-correlated targeting genes with CNV, and then utilized the “enrichKEGG” function for the KEGG pathway enrichment analysis. The cell cycle pathway data was provided. **a** -1og (*P* value) for CNV; **b** gene expression for FC (fold change).

**Additional file 6: Figure S5.** Waterfall plot and analysis regarding the relationship between gene expression and mutation status of top 15 mutated genes. **a** Top 15 genes of mutation frequency, such as *TTN*, *TP53*, *CTNNB1*, *MUC16*, and *ALB*, were selected for the waterfall plot with clinical grading information. **b** Correlation between gene expression and mutation in normal and different pathologic stages of HCC was analyzed.

**Additional file 7: Figure S6.** Waterfall plot and analysis regarding the relationship between gene expression and mutation status of 18 target genes. **a** Fifteen target genes, including *CENPF*, *ASPM*, *SND1*, *MELK*, *TOP2A*, *IQCA1*, *CUZD1*, *TTK*, *NCAPQ*, *FPR2*, *GADD45G*, *CDC20*, *PPBP*, *PCSK1*, *IL11*, *GNGT1*, *CCNE2*, *BDKRB1*, were selected for the waterfall plot with clinical grading information. **b** Correlation between the mutation and expression level of the above genes was analyzed.

**Additional file 8: Figure S7.** Survival curve analysis for mutated *CTNNB1* or *TP53* in different pathologic stages of HCC. The “survminer” R package was used to perform the survival curve analysis for the mutation of **a-d** *CTNNB1*, **e-h** *TP53* in the overall HCC, stage I, II, III-IV of HCC, respectively.

**Additional file 9: Figure S8.** Survival curve analysis for mutated *TTN* or *OBSCN* in different pathologic stages of HCC. Survival curve analyses for the mutation of **a-d** *TTN*, **e-h** *OBSCN* in the overall HCC, stage I, II, III-IV of HCC, were performed by a “survminer” R package, respectively.

**Additional file 10: Figure S9.** Relationship between gene expression and SNP status of *CTNNB1* in different pathologic stages of HCC**. a** We extracted the SNP data of HCC, and identified the SNPs with relatively high frequency. **b** SNP status of *CTNNB1* in the HCC cases. **c** Correlation between gene expression and *CTNNB1* rs121913396, rs121913400, rs121913407, and *TP53* rs28934571 SNP in normal and different pathologic stages of HCC was analyzed.

**Additional file 11: Figure S10.** Survival curve analysis for *CTNNB1* and *TP53* SNPs in different pathologic stages of HCC. The “survminer” R package was used to perform the survival curve analysis for **a** *CTNNB1* rs121913396, **b** rs121913400, **c** rs121913407, and **d** *TP53* rs28934571 SNP in the different pathologic stages of TCGA HCC patients.

**Additional file 12: Figure S11.** Decision tree and random forest analyses for normal controls and HCC cases. We perform a random forest modeling analysis to distinguish the normal controls and HCC cases. **a** Multiple dimension scale plot, **b** ROC curve, and **c-d** largely contributed genes were provided. **e-f** We performed a decision tree modeling analysis and compared the expression of these genes in 50 HCC tissues with adjacent non-tumor tissues, targeting *ECM1*, *FCN2*, *ANGPTL6*, *OIT3*, *ADAMTS13,* and *LRRC14* genes.

**Additional file 13: Figure S12.** Expression level of *SNRPA* in five GEO datasets. From the GEO database, we obtained five independent datasets, including **a** GSE102079 (n = 91), **b** GSE76427 (n = 52), **c** GSE64041 (n = 60), **d** GSE121248 (n = 37), **e** GSE84005 (n = 38), to analyze the expression difference of *SNRPA* between HCC and adjacent non-tumor tissues. Each vertical line represents one patient with HCC.

**Additional file 14: Figure S13.** Expression level of *SNRPD2* in five GEO datasets**.** From the GEO database, we obtained five independent datasets, including **a** GSE102079 (n = 91), **b** GSE76427 (n = 52), **c** GSE64041 (n = 60), **d** GSE121248 (n = 37), **e** GSE84005 (n = 38), to analyze the expression difference of *SNRPD2* between HCC and adjacent non-tumor tissues. Each vertical line represents one patient with HCC.

**Additional file 15: Figure S14.** *GAS2L3* expression analysis of DEN-induced HCC mouse model**.** Based on the tumor and adjacent non-tumor tissues (n = 14) of 20 mg/kg DEN-induced HCC model in mice, qPCR assay was performed to detect the expression level of *GAS2L3*. A wilcox.test was performed, and the results were visualized by a “‘ggplot2’” R package.

**Additional file 16: Figure S15.** Expression level of *SEMA3F* in five GEO datasets. From the GEO database, we obtained five independent datasets, including **a** GSE102079 (n = 91), **b** GSE76427 (n = 52), **c** GSE64041 (n = 60), **d** GSE121248 (n = 37), **e** GSE84005 (n = 38), to analyze the expression difference of *SEMA3F* between HCC and adjacent non-tumor tissues. Each vertical line represents one patient with HCC.

## Data Availability

The datasets generated and/or analyzed in our study are partly available in the TCGA (http://tcga-data.nci.nih.gov/tcga/), GEO (https://www.ncbi.nlm.nih.gov/geo/), and HPA (https://www.proteinatlas.org/about) databases. Others are available from the corresponding author on reasonable request.

## Abbreviations

HCC: Hepatocellular carcinoma
PCA: Principal component analysis
DEN: Diethylnitrosamine
TCGA: The Cancer Genome Atlas
CNV: Copy number variation
SNV: Simple nucleotide variation
SNP: Single nucleotide polymorphism
GEO: Gene Expression Omnibus
NCBI: National Center for Biotechnology Information
LIHC: Liver hepatocellular carcinoma
KM: Kaplan-Meier
log 2: Logarithm base 2
DAVID: Database for annotation, visualization and integrated discovery
GEPIA2: Gene expression profiling and interactive analyses, version two
GO: Gene Ontology
KEGG: Kyoto Encyclopedia of Genes and Genomes
MCODE: Molecular Complex Detection
ROC: Receiver operating characteristic
AUC: Area under the ROC curve
PC: Principal component
qPCR: Quantitative real-time PCR
OS: Overall survival
DFS: Disease free survival
HR: Hazards ratio
GAS2: Growth arrest-specific 2
FC: Fold change
