# Supplementary material for: Genetic expression and mutational profile analysis in different pathologic stages of hepatocellular carcinoma patients
Source: BMC Cancer. 2021 Jul 8;21:786. doi: 10.1186/s12885-021-08442-y (PMC8268469; doi:10.1186/s12885-021-08442-y)
Supplement: Supplementary file 1 — Additional file 1: Table S1. Clinical characteristics of HCC cases in HLivH060PG02 cohort. [file 12885_2021_8442_MOESM1_ESM.docx]

**Table S1. Clinical characteristics of HCC cases in HLivH060PG02 cohort.**

| **Characteristics** | | N |
| --- | --- | --- |
| **Total** | | 30 |
| **Gender** | | |
|  | Male | 23 |
|  | Female | 7 |
| **Age** | | |
|  | <=50 | 8 |
|  | >50 | 22 |
| **Pathologic stage** | | |
|  | Stage I | 1 |
|  | Stage II | 11 |
|  | Stage III | 17 |
|  | Stage IV | 1 |
| **Pathological T** | | |
|  | T2 | 5 |
|  | T3 | 6 |
|  | T4 | 1 |
| **Pathological M** | | |
|  | M0 | 29 |
|  | M1 | 1 |
| **Pathological N** | | |
|  | N0 | 30 |
| **AJCC stage** | | |
|  | 2 | 5 |
|  | 3 | 6 |
|  | 4 | 1 |
| **Vascular invasion** | | |
|  | absent | 11 |
| **Disease history** | | |
|  | Hepatitis B | 15 |
|  | Cirrhosis | 13 |

N, number; AJCC, American Joint Committee on Cancer.
