# Supplementary figures and images for: Genetic expression and mutational profile analysis in different pathologic stages of hepatocellular carcinoma patients
Source: BMC Cancer. 2021 Jul 8;21:786. doi: 10.1186/s12885-021-08442-y (PMC8268469; doi:10.1186/s12885-021-08442-y)

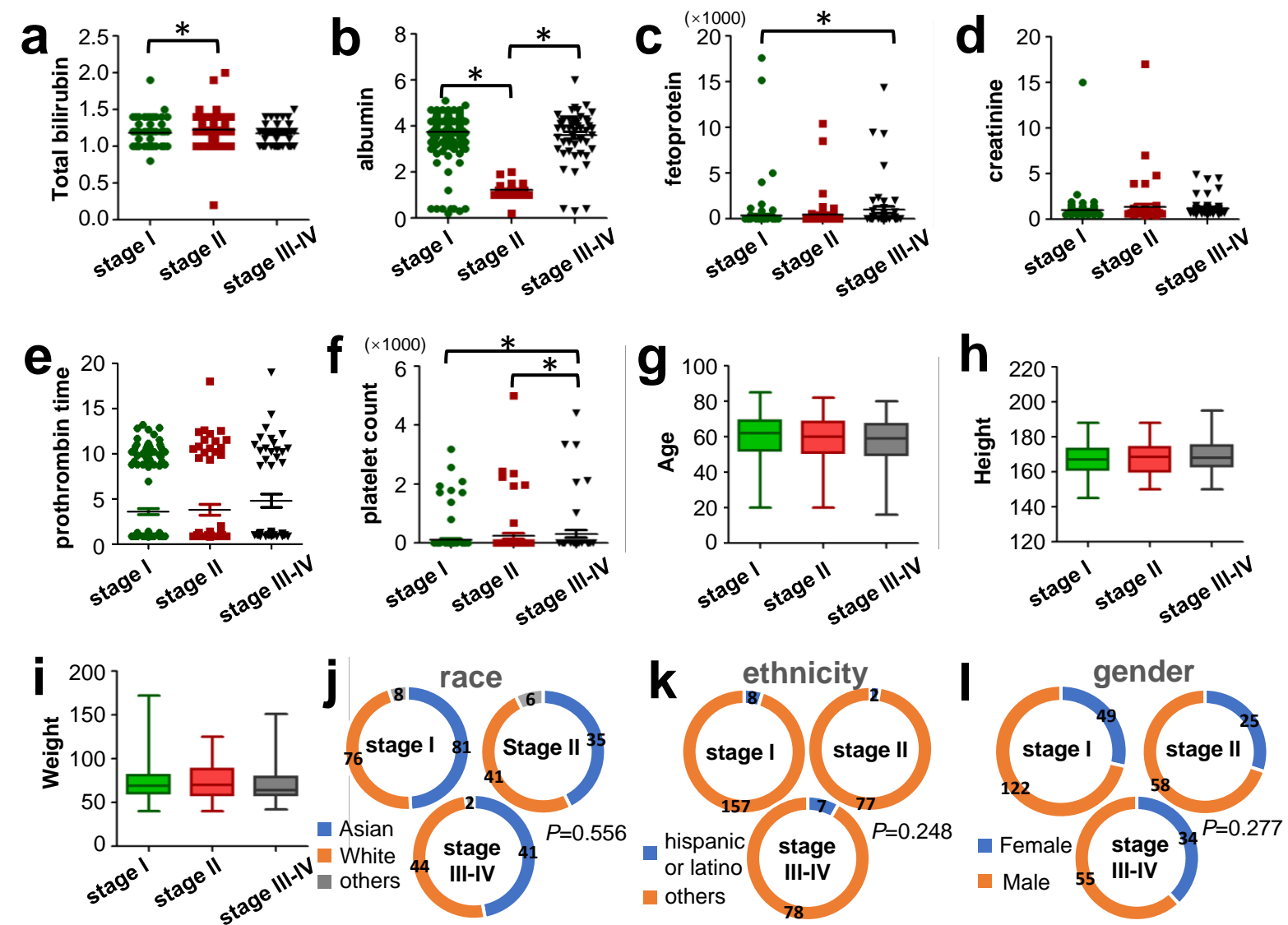

**Figure S1**

Supplement: Supplementary file 2 — Additional file 2: Figure S1. Correlation between clinical or distribution characteristics and different pathologic stages of HCC. We performed the Kruskal-Wallis tests to analyze the relationship between pathologic stage I, II, III-IV, and a total bilirubin, b albumin, c fetoprotein, d creatine, e protherombin time, f platelet count, g age, h height and i weight, respectively. We also performed the chi-square tests to analyze the association between the factors of the j race, k ethnicity, l gender, and pathologic stage I, II, III-IV. * P < 0.05. [file 12885_2021_8442_MOESM2_ESM.pdf]

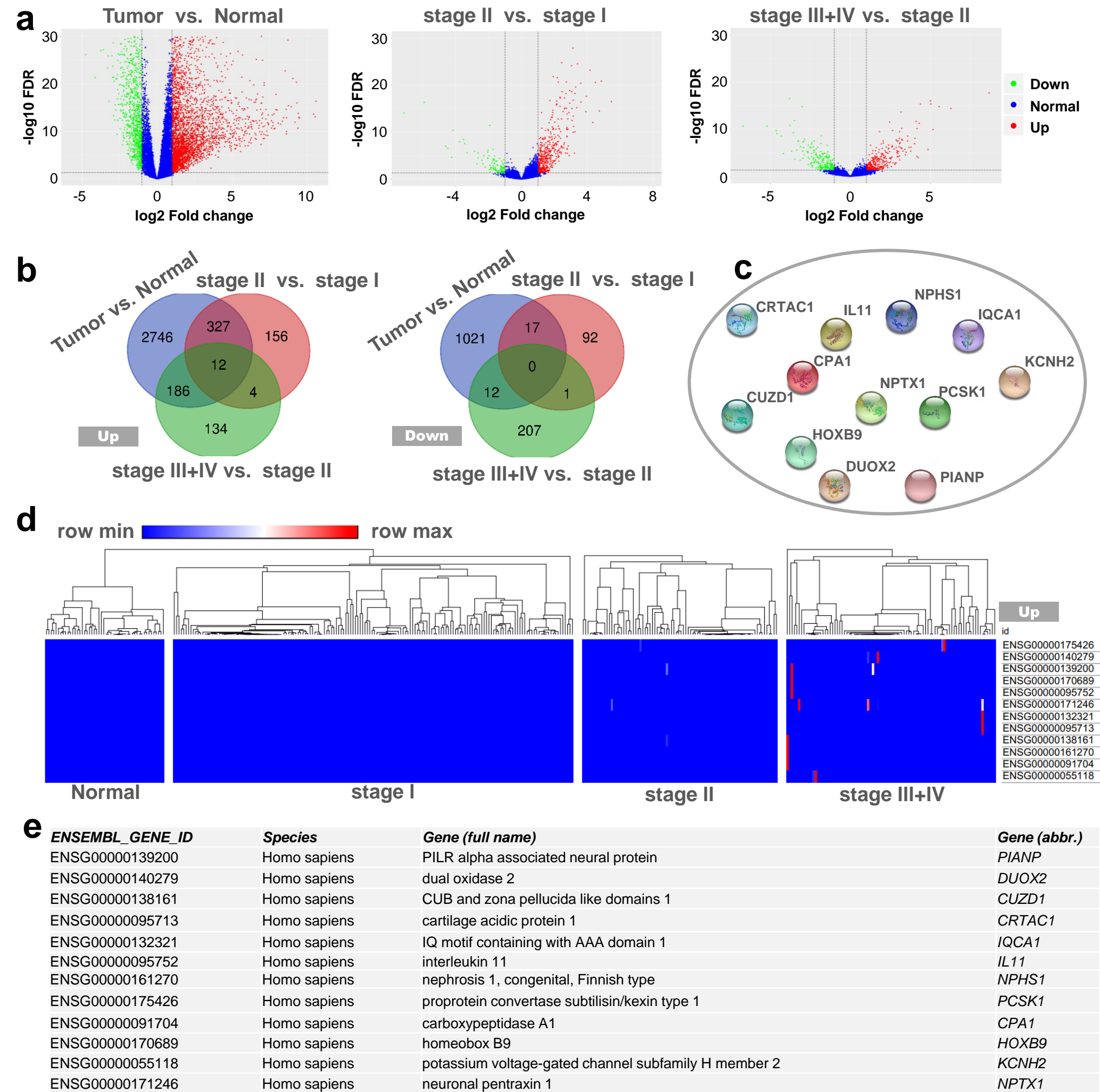

**Figure S2**

Supplement: Supplementary file 3 — Additional file 3: Figure S2. Genetic difference analysis for different pathologic stages of HCC. a Volcano plots of Tumor vs. Normal, stage II vs. stage I, stage III + IV vs. stage II. b Intersection analysis of the above comparisons. c Protein-protein interaction network analysis of intersected genes. d A heat map of cluster analysis. e Full name information of the intersected genes. [file 12885_2021_8442_MOESM3_ESM.pdf]

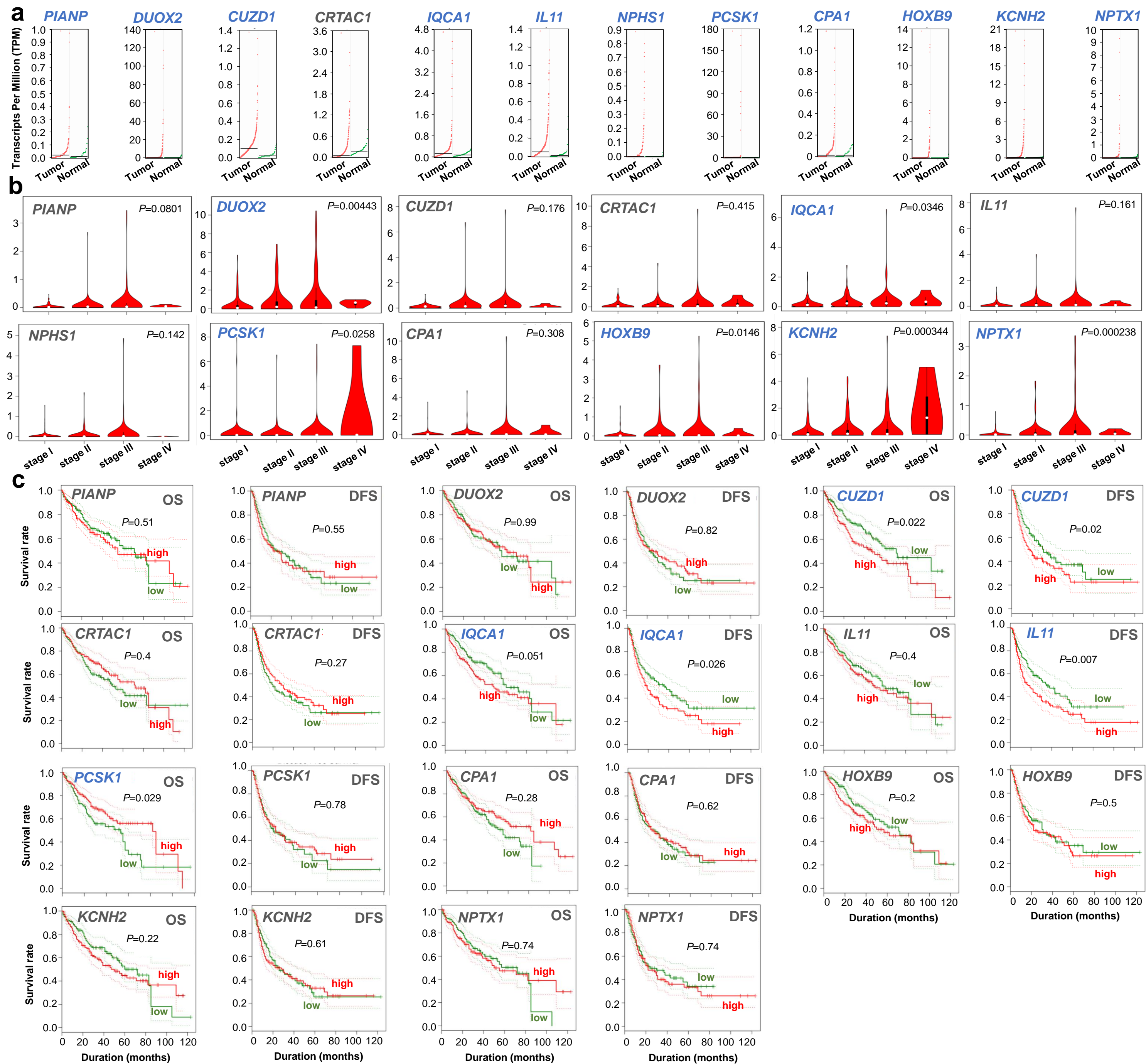

Supplement: Supplementary file 4 — Additional file 4: Figure S3. Expression and relative survival curve analyses for some targeting genes. a We analyzed the expression levels of PIANP, DUOX2, CUZD1, CRTAC1, IQCA1, IL11, NPHS1, PCSK1, CPA1, HOXB9, KCNH2 and NPTX1 genes in a normal and overall HCC, and b different pathologic stages by GEPIA2. c We also performed the Kaplan-Meier estimates of OS or DFS, according to the expression level. [file 12885_2021_8442_MOESM4_ESM.pdf]

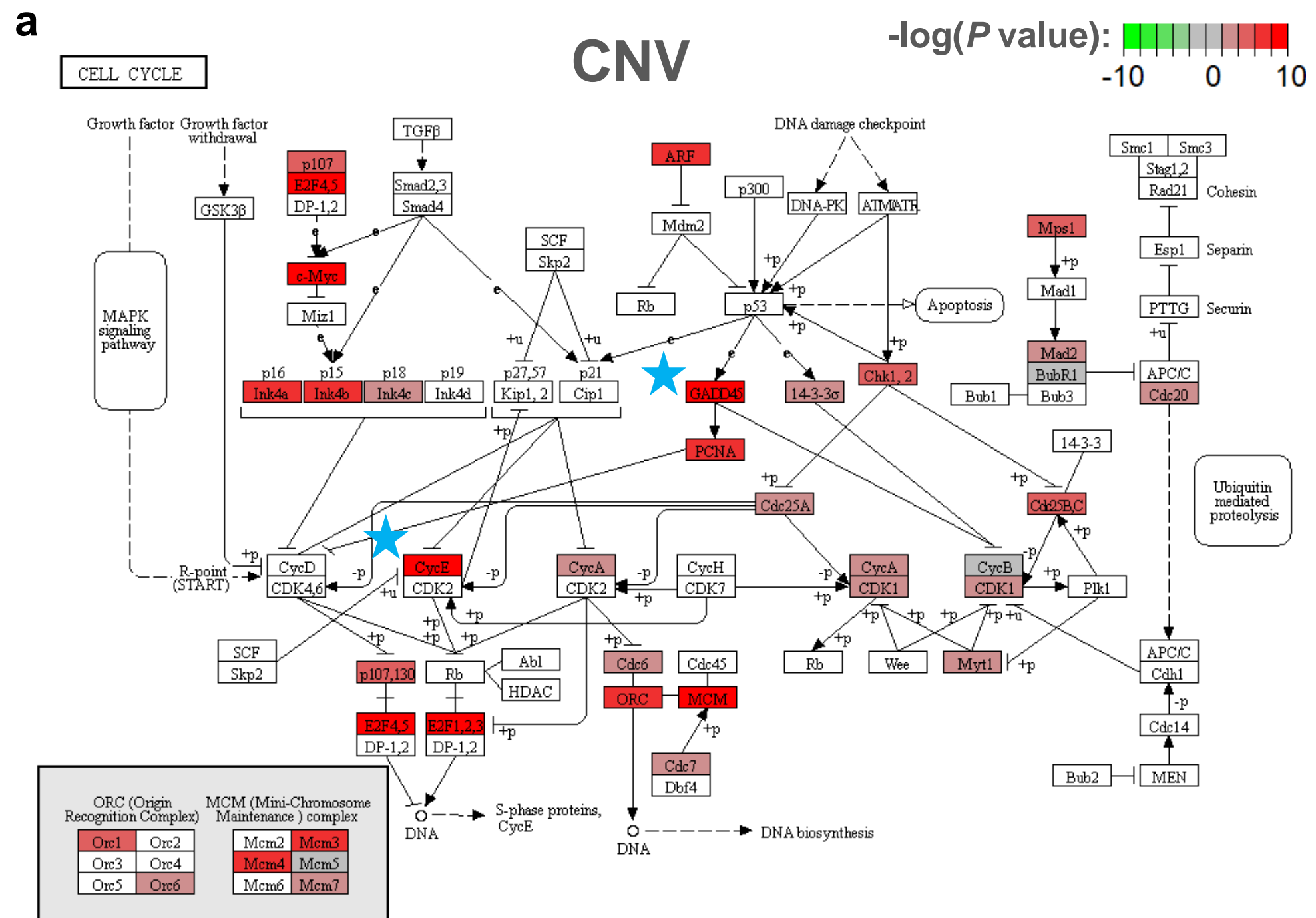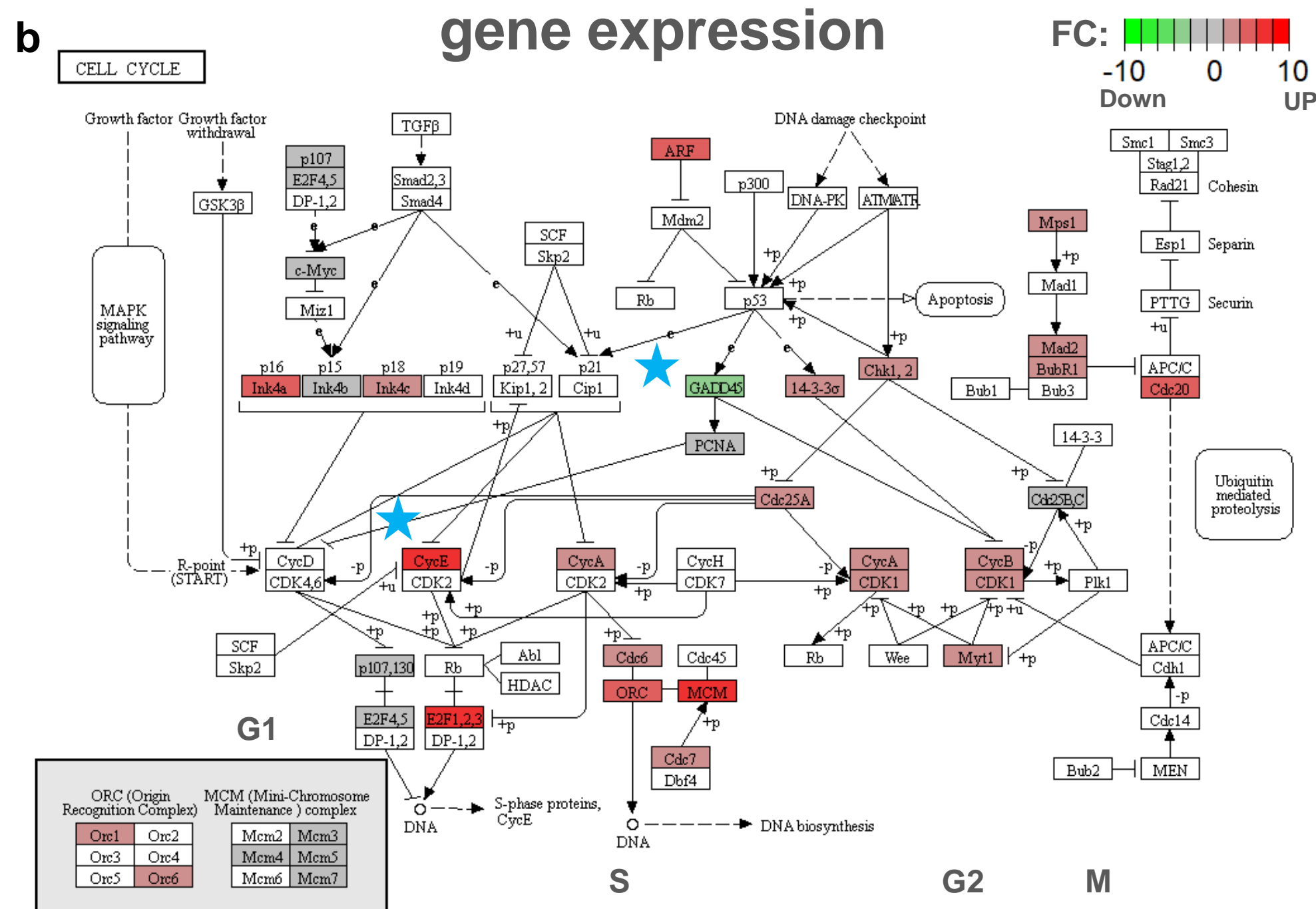

**Figure S4**

Supplement: Supplementary file 5 — Additional file 5: Figure S4. Comparison between CNV and expression level of genes within cell cycle pathway. We performed a Kolmogorov-Smirnov test for correlation analysis to identify the expression-correlated targeting genes with CNV, and then utilized the “enrichKEGG” function for the KEGG pathway enrichment analysis. The cell cycle pathway data was provided. a -1og (P value) for CNV; b gene expression for FC (fold change). [file 12885_2021_8442_MOESM5_ESM.pdf]

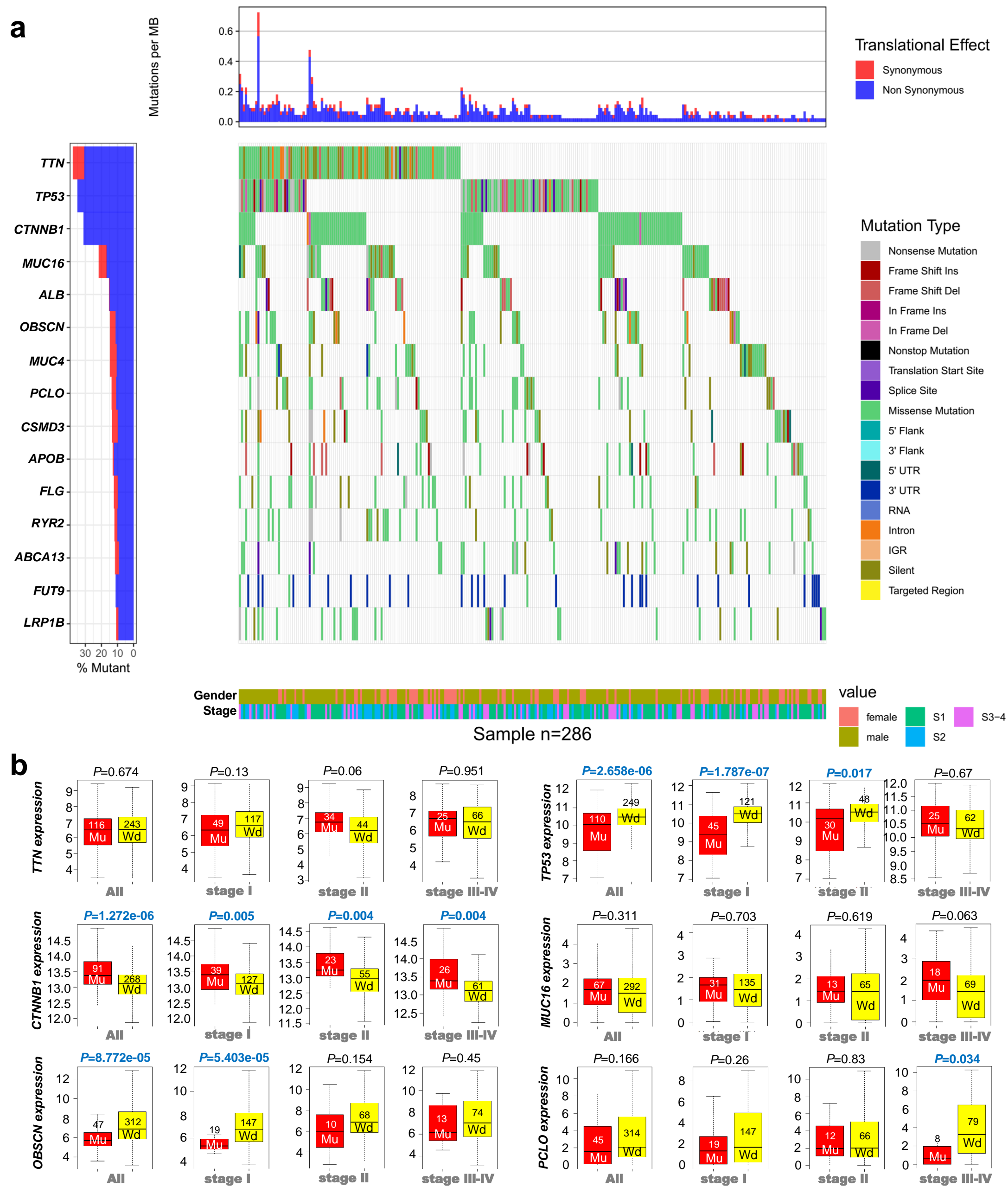

**Figure S5**

Supplement: Supplementary file 6 — Additional file 6: Figure S5. Waterfall plot and analysis regarding the relationship between gene expression and mutation status of top 15 mutated genes. a Top 15 genes of mutation frequency, such as TTN, TP53, CTNNB1, MUC16, and ALB, were selected for the waterfall plot with clinical grading information. b Correlation between gene expression and mutation in normal and different pathologic stages of HCC was analyzed. [file 12885_2021_8442_MOESM6_ESM.pdf]

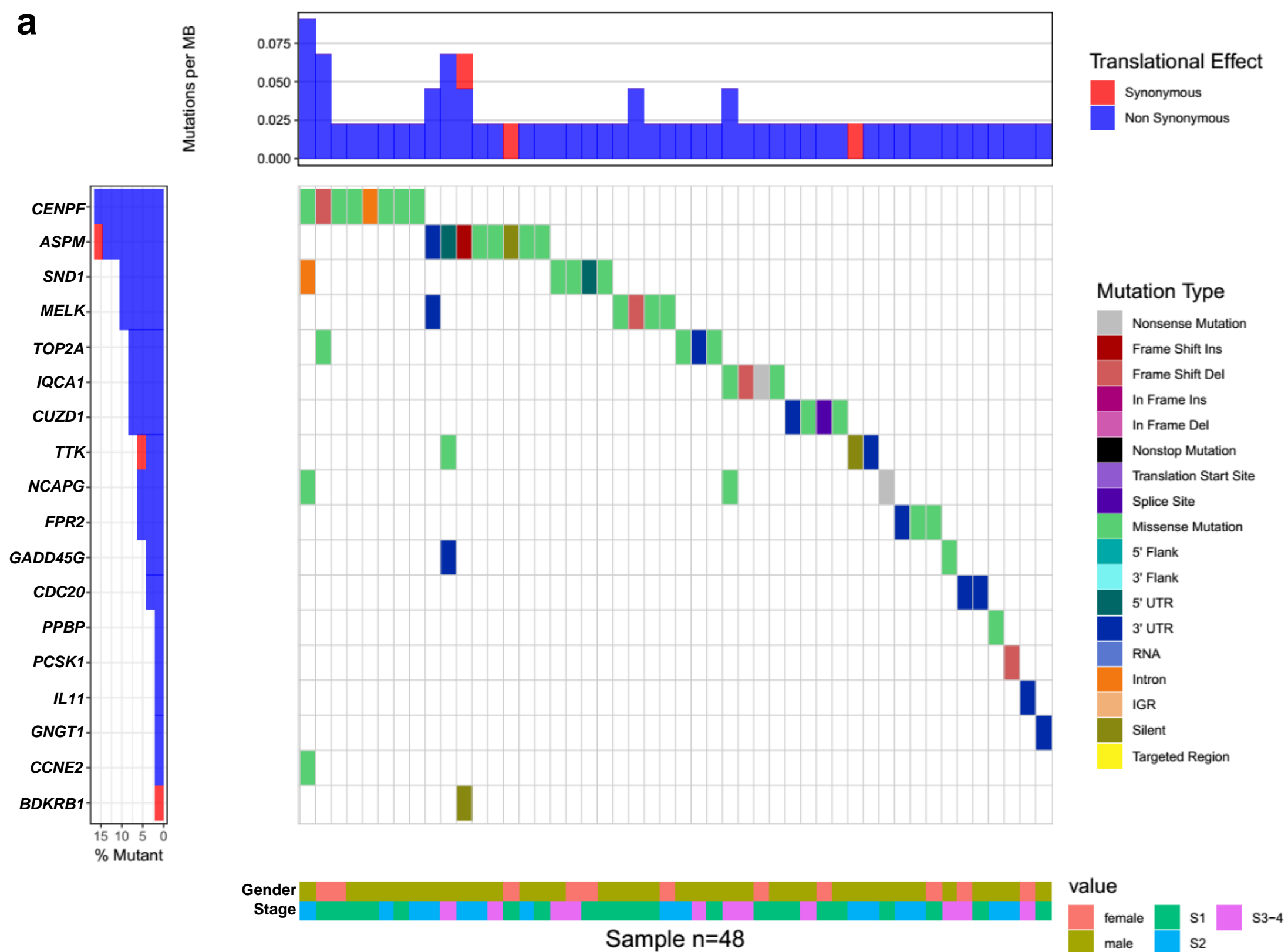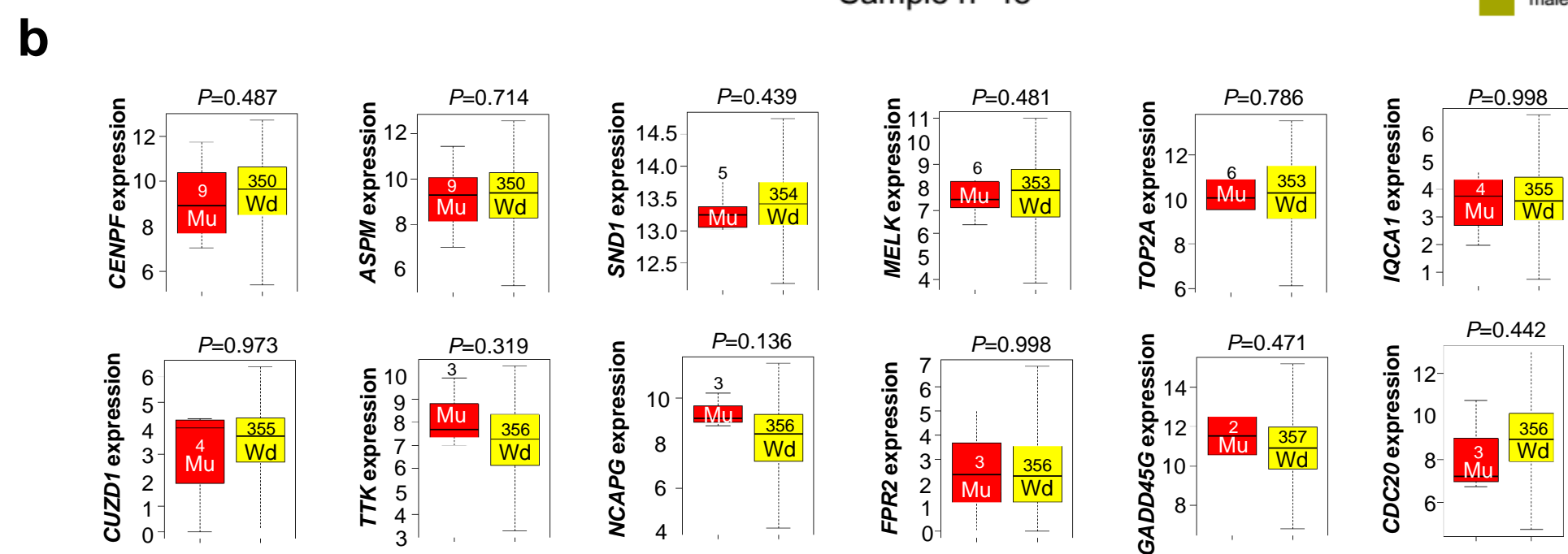

**Figure S6**

Supplement: Supplementary file 7 — Additional file 7: Figure S6. Waterfall plot and analysis regarding the relationship between gene expression and mutation status of 18 target genes. a Fifteen target genes, including CENPF, ASPM, SND1, MELK, TOP2A, IQCA1, CUZD1, TTK, NCAPQ, FPR2, GADD45G, CDC20, PPBP, PCSK1, IL11, GNGT1, CCNE2, BDKRB1, were selected for the waterfall plot with clinical grading information. b Correlation between the mutation and expression level of the above genes was analyzed. [file 12885_2021_8442_MOESM7_ESM.pdf]

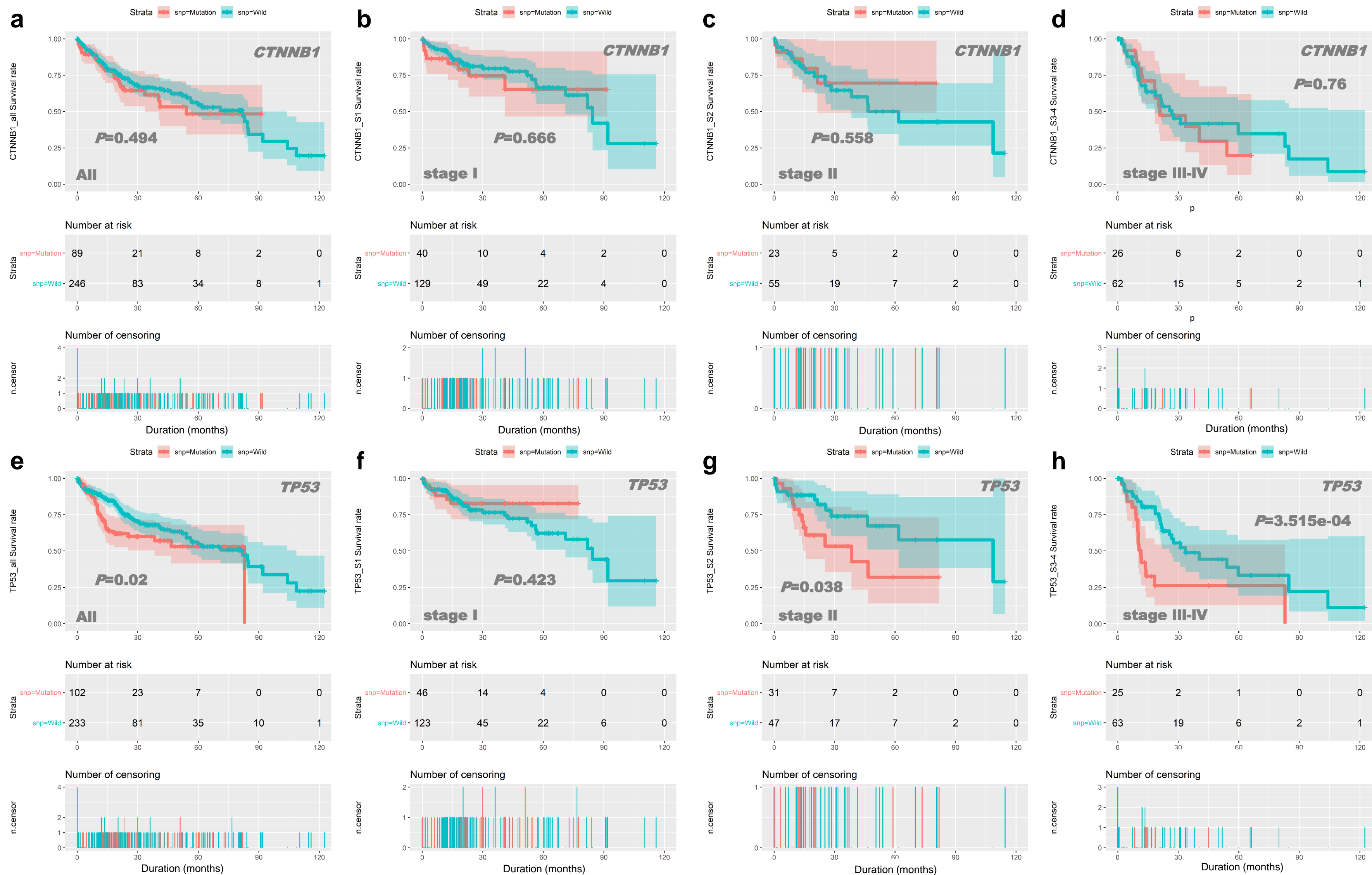

**Figure S7**

Supplement: Supplementary file 8 — Additional file 8: Figure S7. Survival curve analysis for mutated CTNNB1 or TP53 in different pathologic stages of HCC. The “survminer” R package was used to perform the survival curve analysis for the mutation of a-d CTNNB1, e-h TP53 in the overall HCC, stage I, II, III-IV of HCC, respectively. [file 12885_2021_8442_MOESM8_ESM.pdf]

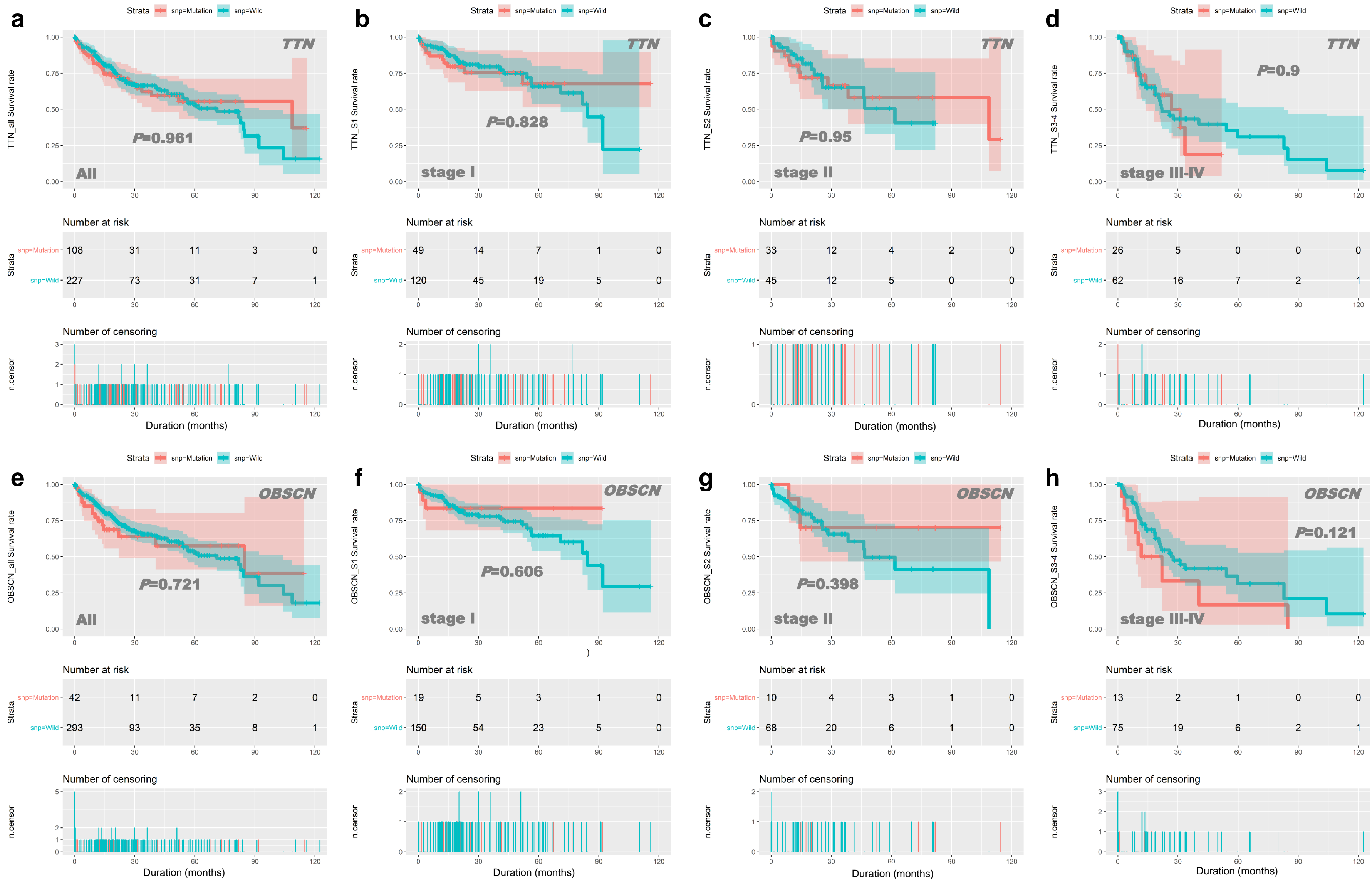

**Figure S8**

Supplement: Supplementary file 9 — Additional file 9: Figure S8. Survival curve analysis for mutated TTN or OBSCN in different pathologic stages of HCC. Survival curve analyses for the mutation of a-d TTN, e-h OBSCN in the overall HCC, stage I, II, III-IV of HCC, were performed by a “survminer” R package, respectively. [file 12885_2021_8442_MOESM9_ESM.pdf]

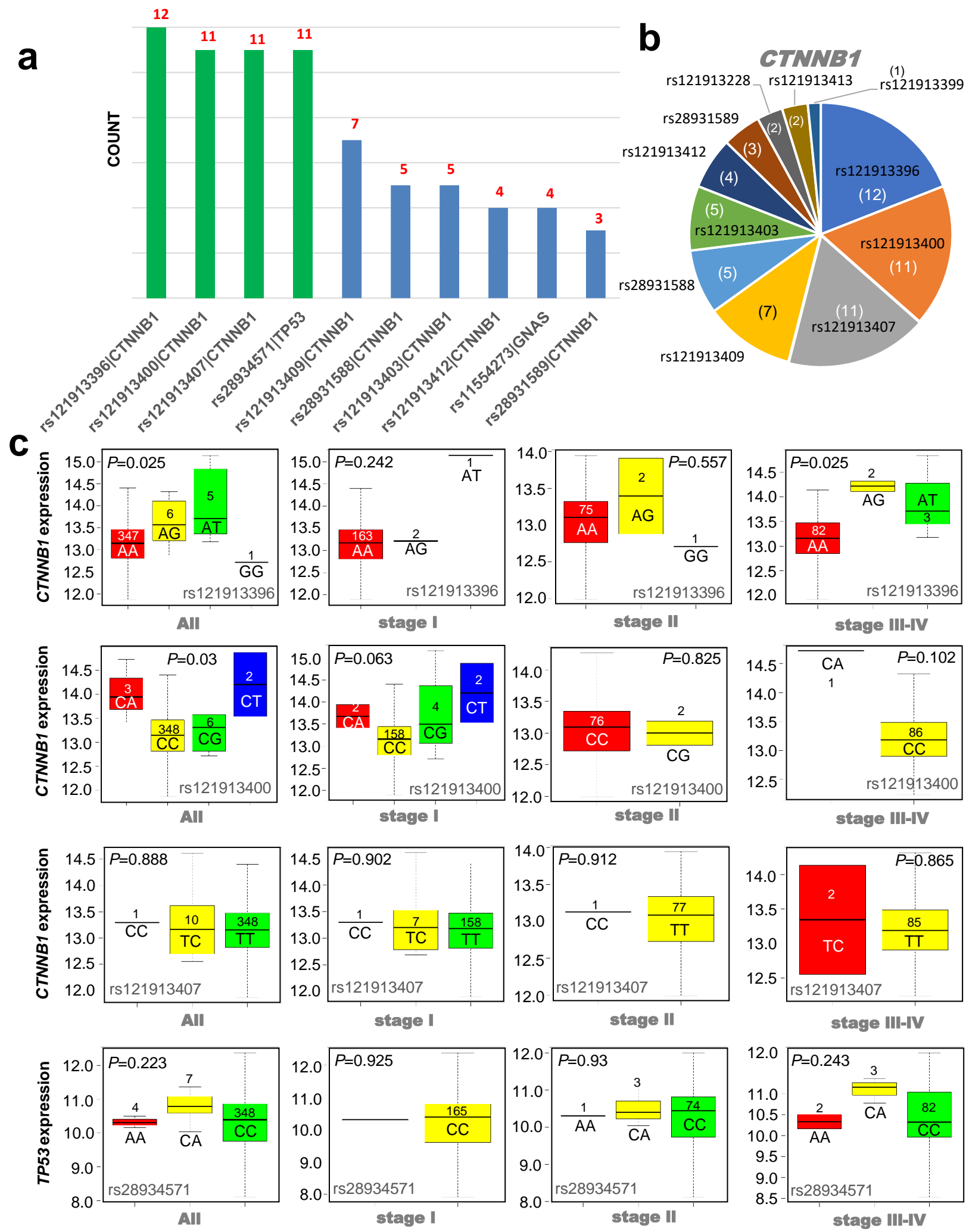

**Figure S9**

Supplement: Supplementary file 10 — Additional file 10: Figure S9. Relationship between gene expression and SNP status of CTNNB1 in different pathologic stages of HCC. a We extracted the SNP data of HCC, and identified the SNPs with relatively high frequency. b SNP status of CTNNB1 in the HCC cases. c Correlation between gene expression and CTNNB1 rs121913396, rs121913400, rs121913407, and TP53 rs28934571 SNP in normal and different pathologic stages of HCC was analyzed. [file 12885_2021_8442_MOESM10_ESM.pdf]

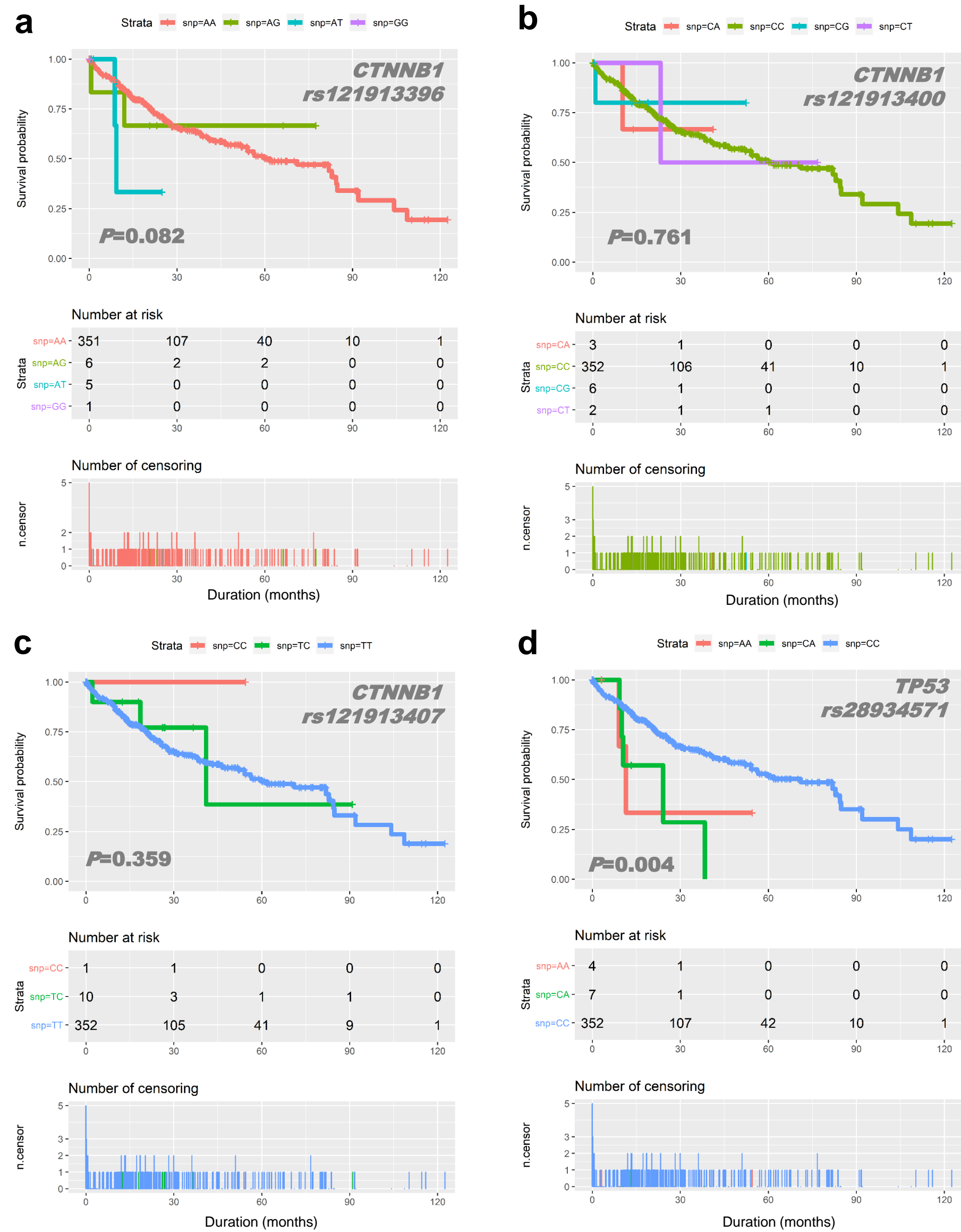

**Figure S10**

Supplement: Supplementary file 11 — Additional file 11: Figure S10. Survival curve analysis for CTNNB1 and TP53 SNPs in different pathologic stages of HCC. The “survminer” R package was used to perform the survival curve analysis for a CTNNB1 rs121913396, b rs121913400, c rs121913407, and d TP53 rs28934571 SNP in the different pathologic stages of TCGA HCC patients. [file 12885_2021_8442_MOESM11_ESM.pdf]

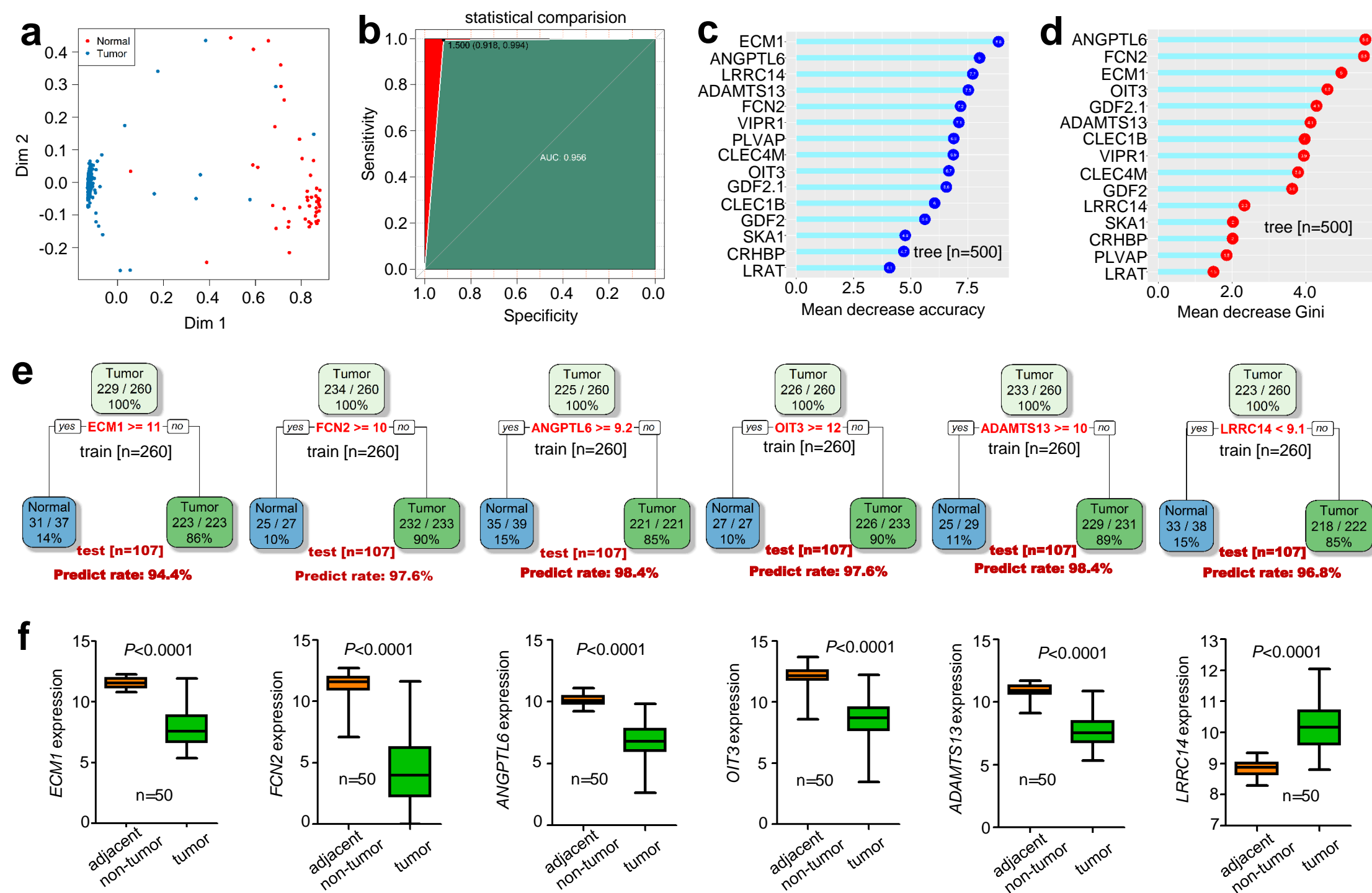

**Figure S11**

Supplement: Supplementary file 12 — Additional file 12: Figure S11. Decision tree and random forest analyses for normal controls and HCC cases. We perform a random forest modeling analysis to distinguish the normal controls and HCC cases. a Multiple dimension scale plot, b ROC curve, and c-d largely contributed genes were provided. e-f We performed a decision tree modeling analysis and compared the expression of these genes in 50 HCC tissues with adjacent non-tumor tissues, targeting ECM1, FCN2, ANGPTL6, OIT3, ADAMTS13, and LRRC14 genes. [file 12885_2021_8442_MOESM12_ESM.pdf]

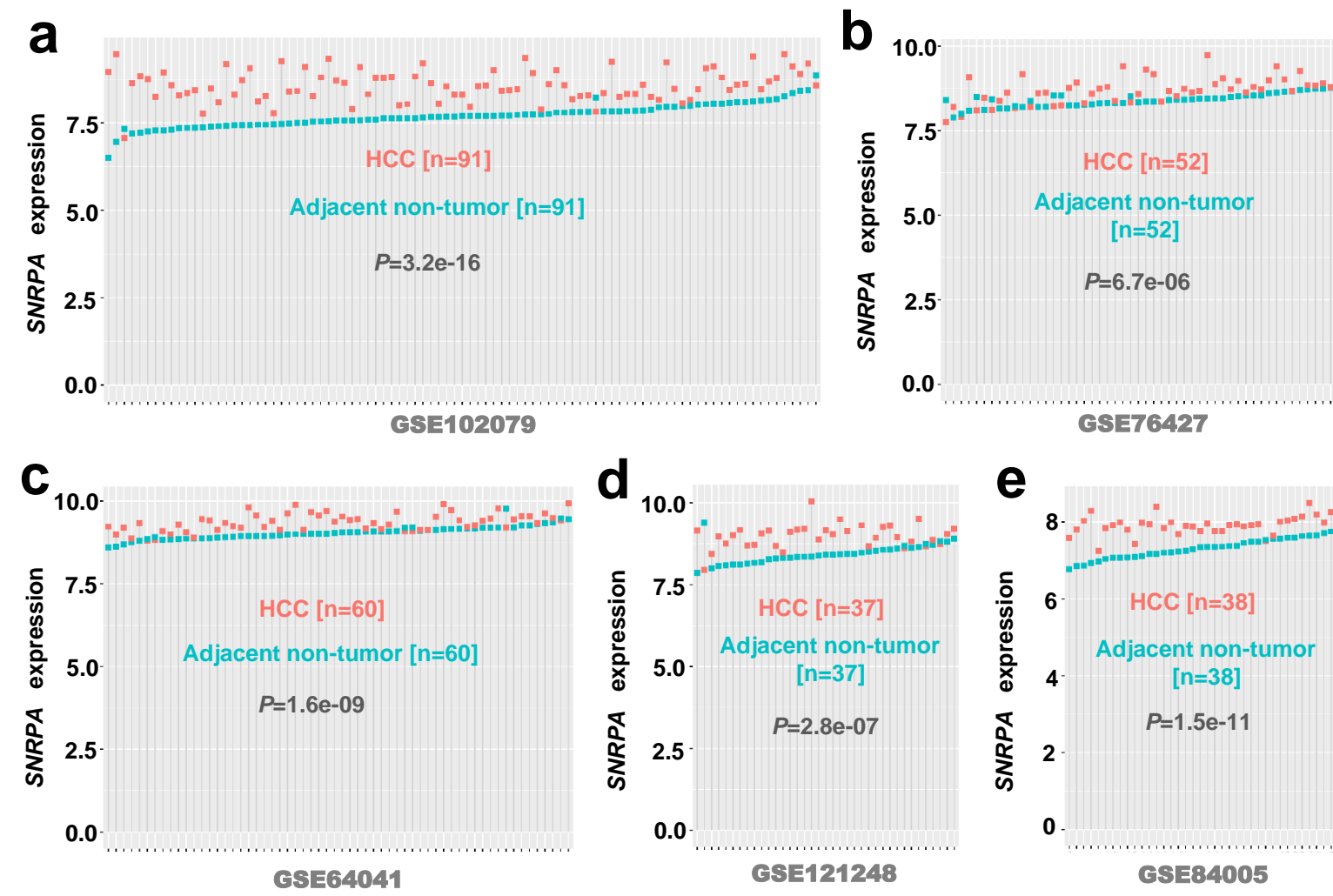

**Figure S12**

Supplement: Supplementary file 13 — Additional file 13: Figure S12. Expression level of SNRPA in five GEO datasets. From the GEO database, we obtained five independent datasets, including a GSE102079 (n = 91), b GSE76427 (n = 52), c GSE64041 (n = 60), d GSE121248 (n = 37), e GSE84005 (n = 38), to analyze the expression difference of SNRPA between HCC and adjacent non-tumor tissues. Each vertical line represents one patient with HCC. [file 12885_2021_8442_MOESM13_ESM.pdf]

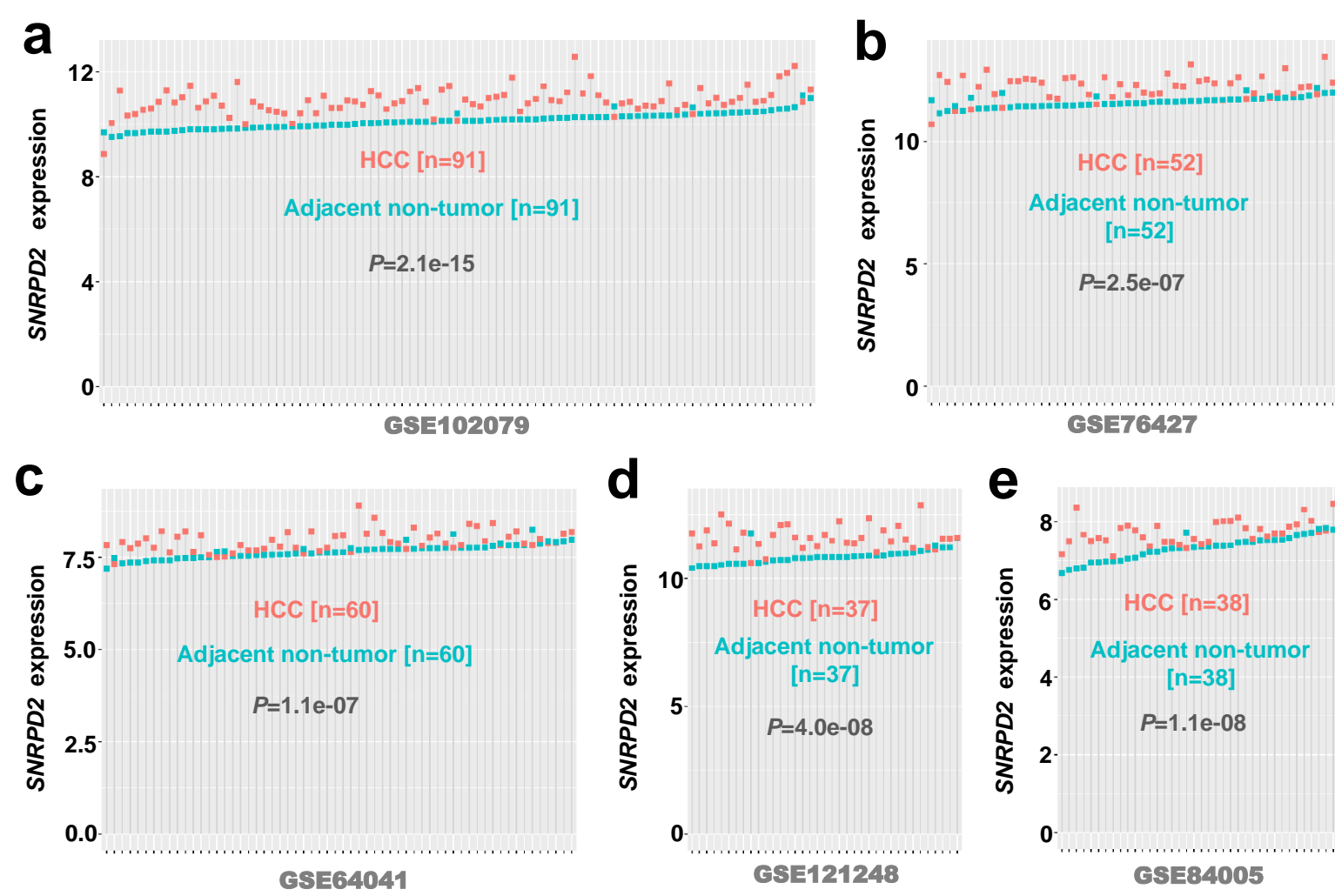

**Figure S13**

Supplement: Supplementary file 14 — Additional file 14: Figure S13. Expression level of SNRPD2 in five GEO datasets. From the GEO database, we obtained five independent datasets, including a GSE102079 (n = 91), b GSE76427 (n = 52), c GSE64041 (n = 60), d GSE121248 (n = 37), e GSE84005 (n = 38), to analyze the expression difference of SNRPD2 between HCC and adjacent non-tumor tissues. Each vertical line represents one patient with HCC. [file 12885_2021_8442_MOESM14_ESM.pdf]

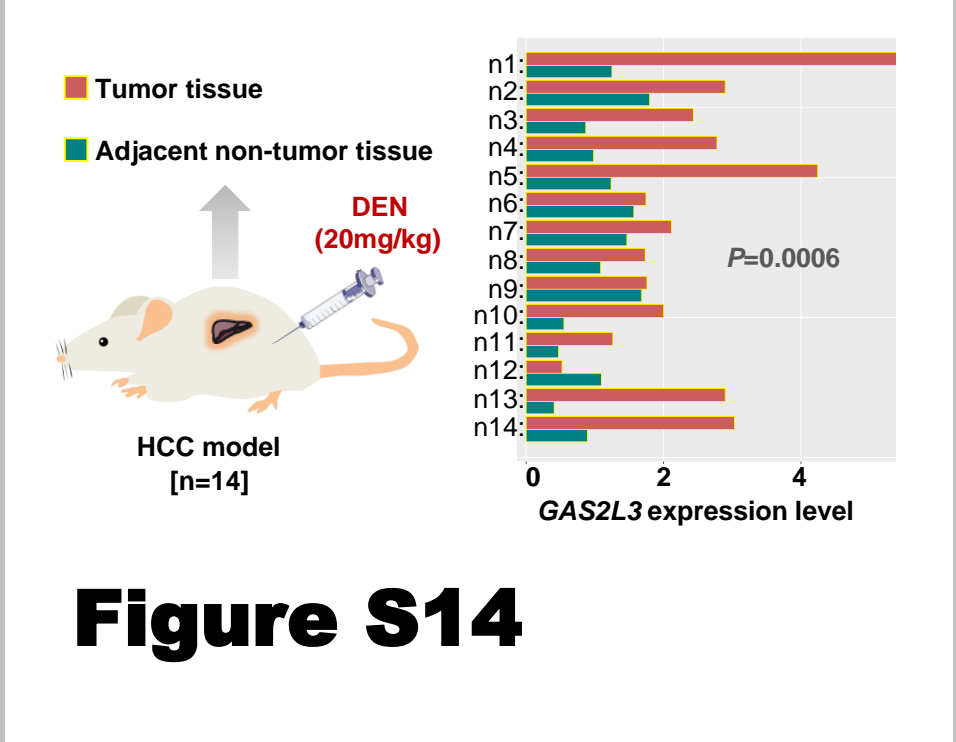

**Figure S14**

Supplement: Supplementary file 15 — Additional file 15: Figure S14. GAS2L3 expression analysis of DEN-induced HCC mouse model. Based on the tumor and adjacent non-tumor tissues (n = 14) of 20 mg/kg DEN-induced HCC model in mice, qPCR assay was performed to detect the expression level of GAS2L3. A wilcox.test was performed, and the results were visualized by a “‘ggplot2’” R package. [file 12885_2021_8442_MOESM15_ESM.pdf]

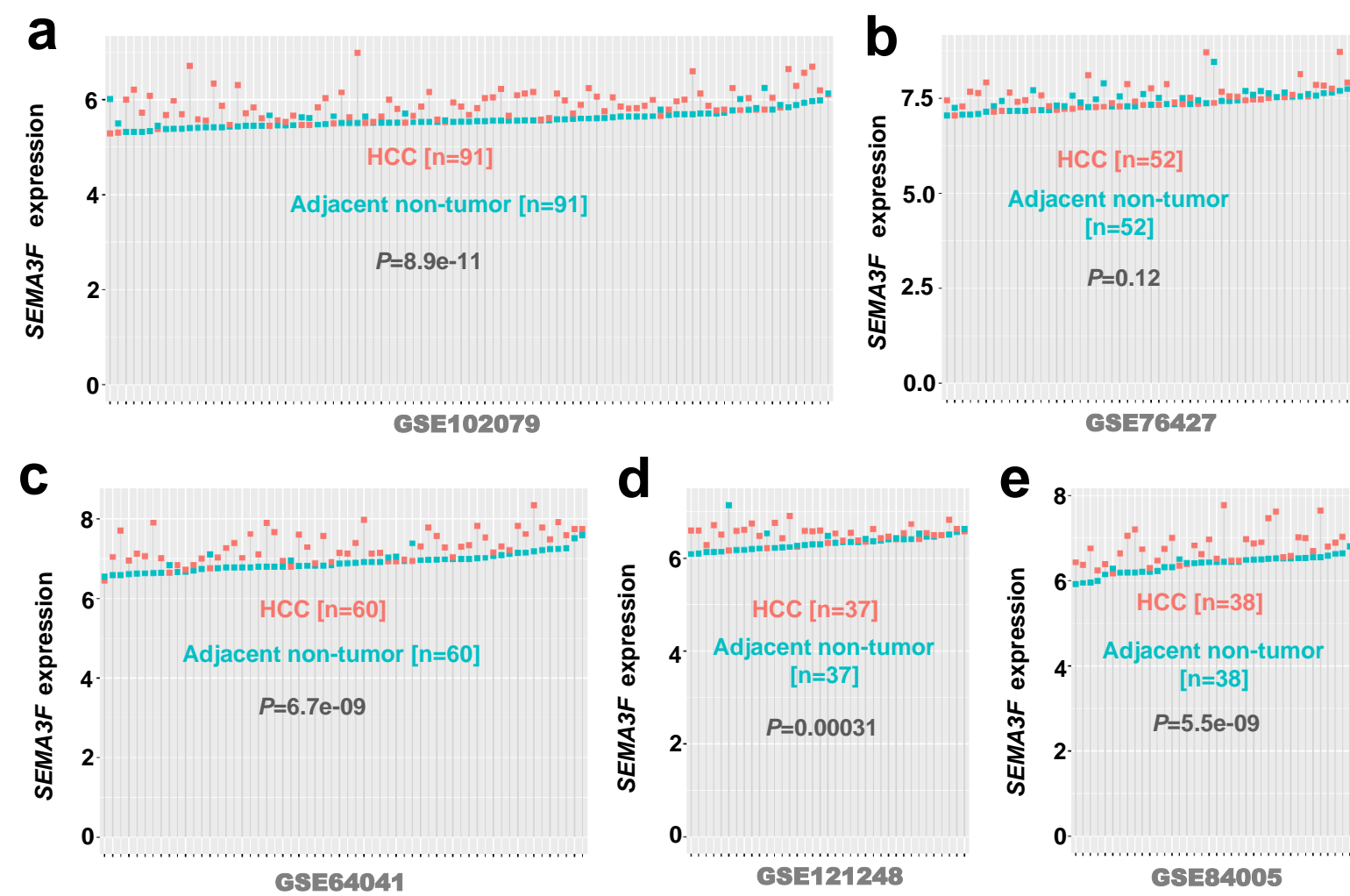

**Figure S15**

Supplement: Supplementary file 16 — Additional file 16: Figure S15. Expression level of SEMA3F in five GEO datasets. From the GEO database, we obtained five independent datasets, including a GSE102079 (n = 91), b GSE76427 (n = 52), c GSE64041 (n = 60), d GSE121248 (n = 37), e GSE84005 (n = 38), to analyze the expression difference of SEMA3F between HCC and adjacent non-tumor tissues. Each vertical line represents one patient with HCC. [file 12885_2021_8442_MOESM16_ESM.pdf]
